# Does the brain’s E:I balance really shape long-range temporal correlations? Lessons learned from 3T MRI

**DOI:** 10.1162/IMAG.a.1252

**Published:** 2026-05-29

**Authors:** Lydia Sochan, Jessica Archibald, Alexander Mark Weber

**Affiliations:** School of Biomedical Engineering, The University of British Columbia, Vancouver, BC, Canada; Department of Radiology, Weill Cornell Medicine, New York, NY, United States; BC Children’s Hospital Research Institute, The University of British Columbia, Vancouver, BC, Canada; Pediatrics, The University of British Columbia, Vancouver, BC, Canada

**Keywords:** Hurst exponent, long range temporal correlation, excitatory/inhibitory balance, criticality, complex systems, visual task, functional magnetic resonance imaging, magnetic resonance spectroscopy, functional magnetic resonance spectroscopy

## Abstract

A 3T multimodal MR study of healthy adults (n = 18; 10 female; 21.3–53.4 years) was performed to investigate the relationship between functional magnetic resonance imaging (fMRI) long-range temporal correlations and excitatory/inhibitory balance (via single voxel magnetic resonance spectroscopy measurements of glutamate and γ-aminobutyric acid (GABA)). The study objective was to determine whether the Hurst exponent (H)—an estimate of self-correlation and signal complexity—of the blood oxygen level-dependent signal was correlated with the excitatory–inhibitory (E:I) ratio. E:I has been proposed to serve as a control parameter for brain criticality—the theory that the brain operates near a critical point between order and disorder, optimizing information processing and adaptability—which H is believed to be a measure of. Thus, understanding whether H and E:I are indeed correlated would clarify this relationship. Moreover, findings in this domain have implications for neurological and neuropsychiatric conditions with disrupted E:I balance, such as autism, schizophrenia, and Alzheimer’s disease. From a practical perspective, H is easier to accurately measure than E:I ratio at 3T MRI. If H can serve as a proxy for E:I, it may serve as a more practical clinical biomarker for this imbalance and for neuroscience research in general. The study collected functional MRI and magnetic resonance spectroscopy data during rest and movie watching. H and E:I (glutamate/GABA) were not found to be correlated. H was found to increase with movie watching compared with rest, while E:I did not change between conditions. This study represents the first attempt to investigate this connection *in vivo* in humans. We conclude that, at 3T and with our particular methodologies, no association was found. We end with lessons learned and suggestions for future research.

## 1 Introduction

The human brain consists of interconnected networks of neurons whose collective activity gives rise to complex behaviours and cognition. In order to understand how neural systems generate this emergent functionality, neuroscience is increasingly using tools and concepts from the science of complexity. Recent advances have led to the emergence of the Critical Brain Hypothesis, which posits that the brain—across all regions and networks—operates at a “critical point”, a state where both order and disorder are balanced to enable the most efficient information processing (Baranger, 2000; Bassett & Gazzaniga, 2011; Baumgarten & Bornholdt, 2019; J. Beggs & Timme, 2012; Bruining et al., 2020; Deco et al., 2013; Gao et al., 2017; Liang et al., 2025; Lombardi et al., 2017; Poil et al., 2012; Rubinov et al., 2011; Tian et al., 2022; Trakoshis et al., 2020; Zimmern, 2020). In fact, criticality is perhaps the main way that nature produces stable complex systems (Bak, 1996; Langton, 1990). When in a critical state, the brain is optimally responsive to both internal and external stimuli while maintaining a balance between stability and instability (J. Beggs & Timme, 2012; Deco et al., 2013; Rubinov et al., 2011; Tian et al., 2022).

Currently, the most popular theory proposing how the brain achieves this delicate balance—what is known as the “control parameter”—is the excitation–inhibition (E:I) ratio: the balance of excitatory and inhibitory neural activity, often operationalized as the ratio of the primary excitatory and inhibitory neurotransmitters (i.e., glutamate (Glu) and γ-aminobutyric acid (GABA)) (Baumgarten & Bornholdt, 2019; Bruining et al., 2020; Gao et al., 2017; Liang et al., 2025; Lombardi et al., 2017; Trakoshis et al., 2020). This theory essentially relies on the fact that E:I is a primary driver of the signal-to-noise ratio (SNR) of neuronal signalling, and hence could drive transitions between order and disorder (Liang et al., 2025; Rubenstein & Merzenich, 2003). Thus, the E:I theory of criticality posits that altering the E:I ratio would allow for movement between sub- and super-critical states (Liang et al., 2025). When excitation is perfectly balanced with inhibition, the system is at a critical point, but if either excitation or inhibition is favoured, the system can become overly noisy (excess excitation) or overly controlled (excess inhibition), respectively (Liang et al., 2025). Thus, the relationship between E:I balance and large-scale brain dynamics offers a promising link between synaptic physiology and macroscopic neural computation, and for elucidating fundamental neural computational principles. Furthermore, understanding this relationship could hold great clinical relevance, as disruptions in E:I homeostasis have been implicated in multiple neuropsychiatric and neurodevelopmental disorders—including autism, schizophrenia, and Alzheimer’s disease—making this an important target for biomarker development and therapeutic intervention (Bruining et al., 2020; Lauterborn et al., 2021; Sohal & Rubenstein, 2019).

*In vivo* measurement of brain criticality can be achieved using several available methods, as systems near a critical state often exhibit fractal-like fluctuations or scale invariance (Muñoz, 2018). Scale invariance has been observed extensively across various neuronal spatio-temporal scales, from dendritic branching structures (Caserta et al., 1995) in the spatial domain to neurotransmitter release (Lowen et al., 1997), neuronal firing rates (Mazzoni et al., 2007), local field potentials (Bédard & Destexhe, 2009), electroencephalography (EEG) (E. T. Bullmore et al., 1994), and functional magnetic resonance imaging (fMRI)—which measures the blood oxygen level-dependent (BOLD) signal (O. L. Campbell & Weber, 2022; Fadili & Bullmore, 2002; Zarahn et al., 1997)—in the time domain. This fractal structure can be quantified by assessing the signal’s long-range temporal correlations (LRTC), which reflect the persistence or memory in a time series, where future values are statistically related to past values over long extended periods. The Hurst exponent (H) is a useful measure for evaluating LRTC (O. L. Campbell & Weber, 2022; Eke et al., 2002). An H value between 0.5 and 1 indicates a long-range positive correlation, with large values being followed by large values and small values by small ones. Conversely, an H exponent value between 0 and 0.5 indicates a long-range negative correlation, where large values are likely to be followed by small ones and vice versa. An H value of 0.5 is uncorrelated random noise (Hurst, 1951).

H has emerged as a valuable tool in neuroscience and clinical research. Typically, H values reported in adult brains are above 0.5, with higher H values in grey matter than in white matter or cerebrospinal fluid (Dong et al., 2018; Wink et al., 2008). Some key findings from neuroscience research include a decrease in H during task performance (Ciuciu et al., 2014; He, 2011); an increase in H during movie watching in the visual resting-state network (O. Campbell et al., 2022); negative correlations with task novelty and difficulty (Churchill et al., 2016); increases with age in the frontal and parietal lobes (Dong et al., 2018), and hippocampus (Wink et al., 2006); decreases with age in the insula, and limbic, occipital and temporal lobes (Dong et al., 2018); and H <0.5 in preterm infants (Mella et al., 2024). For a more in-depth review, see O. L. Campbell and Weber (2022). In terms of clinical findings, abnormal H values have been identified in Alzheimer’s disease (AD) (Maxim et al., 2005; Warsi et al., 2012), autism (Dona, Hall, et al., 2017; Lai et al., 2010; Linke et al., 2024; Uscătescu et al., 2023), mild traumatic brain injury (Dona, Noseworthy, et al., 2017), major depressive disorder (Jing et al., 2017; Wei et al., 2013), and schizophrenia (Sokunbi et al., 2014; Uscătescu et al., 2023). Crucially, these same disorders have been associated with imbalances in E:I (Bruining et al., 2020; Kang et al., 2019; Lauterborn et al., 2021; Page & Coutellier, 2019; Sohal & Rubenstein, 2019; Uzunova et al., 2016; Vico Varela et al., 2019).

In addition to its implications for the critical brain hypothesis, establishing a connection between E:I and H could facilitate simpler estimation of excitatory and inhibitory neurotransmitters, as precise measurement of E:I is technically challenging (Ajram et al., 2019). Currently, magnetic resonance spectroscopy (MRS) is the only non-invasive method for *in vivo* assessment of the Glu/GABA ratio (excitatory to inhibitory neurotransmitters) in humans (Harris et al., 2017; Stanley & Raz, 2018). Unfortunately, MRS suffers from limited spatial and temporal resolution, with single-voxel spectrosopy sizes between 20 and 27 cm^3^, and scan times between 5 and 10 minutes (Ajram et al., 2019; Gao et al., 2017; Stanley & Raz, 2018). In addition, quantification of GABA at 3T necessitates the use of a specialized J-difference spectral-editing sequence (such as MEGA-PRESS), in order to isolate GABA (at 3 ppm, 2.3 ppm, and 1.9 ppm) from overlapping peaks (e.g., creatine and glutamate) (Peek et al., 2023). The need for this additional sequence, therefore, reduces the temporal resolution further. If H could function as a substitute for E:I, it could simplify the estimation of E:I.

Beyond clinical observations suggesting that both H and E:I imbalance are altered in neuropsychiatric conditions, several studies have attempted to show that critical-state dynamics emerge when excitation and inhibition are balanced. Indirect evidence comes from pharmacological manipulation of GABAergic neurotransmission, which has been shown to change the strength of LRTC *in vitro* (Poil et al., 2011) and in the beta-frequency range in humans using EEG (Monto et al., 2007), and to eliminate the power-law scaling of neuronal avalanches in rats (J. M. Beggs & Plenz, 2003; Gireesh & Plenz, 2008; Yang et al., 2012). More direct efforts linking H and E:I have mostly relied on computational models or animal studies (Baumgarten & Bornholdt, 2019; Bruining et al., 2020; Gao et al., 2017; Liang et al., 2025; Lombardi et al., 2017; Poil et al., 2012; Trakoshis et al., 2020), and the results remain inconsistent, ranging from positive to negative or even U-shaped associations. These discrepancies may reflect differences in model systems (e.g., animal vs. computer simulations), methodological choices (such as how H or E:I is estimated), or may represent counter evidence against the theory that these measures are systematically related. Indeed, other theories for how the brain maintains criticality exist, such as neuromodulator regulation (Avrămiea, 2023; O’Byrne & Jerbi, 2022; Plenz et al., 2021) or synaptic plasticity (Ikeda et al., 2025; Zeraati et al., 2021), to name a few. In spite of the lack of direct evidence, several recent human studies have used fMRI-derived H as a direct proxy for E:I balance (Fotiadis et al., 2023; Linke et al., 2024; Uscătescu et al., 2023; Xie et al., 2024); yet, no study to date has tested the empirical relationship between H and neurochemical estimates of E:I (e.g., Glu/GABA ratio) *in vivo* in humans.

The theory that E:I serves as the primary control parameter governing H—and, by extension, brain criticality—offers a framework with clear, falsifiable predictions that can be directly tested. Specifically, if this framework holds, E:I and H should covary systematically in the human brain across individuals, age, brain regions, and cognitive states. To empirically test this prediction, the present study examined the E:I–Hurst relationship in the human visual cortex during rest and naturalistic movie watching. Although a single condition would be sufficient to test the correlation between E:I and H, we included two conditions to broaden the dynamic range of neural and neurochemical measures—thereby increasing our sensitivity to detect a correlation—and to provide validation that our metrics behave as expected across qualitatively different brain states. Naturalistic movie watching is known to reliably engage the visual cortex with complex, temporally structured input, making it a useful condition for probing changes in the temporal dynamics of neural activity (O. Campbell et al., 2022; Vanderwal et al., 2019). Prior work from our group has shown that H values increased during movie watching compared with rest (O. Campbell et al., 2022), while work from others has found changes in Glu and GABA with sustained visual stimulation (Kurcyus et al., 2018; Y. Lin et al., 2012; Martínez-Maestro et al., 2019; Saucedo et al., n.d.). We hypothesized that H and Glu would increase during movie watching (O. Campbell et al., 2022; Y. Lin et al., 2012; Martínez-Maestro et al., 2019), that GABA would decrease (Kurcyus et al., 2018; Saucedo et al., n.d.), and that H would be positively correlated with E:I in both conditions (Gao et al., 2017).

## 2 Methods

### 2.1 Participants

Twenty-seven healthy adult participants were recruited to the study. One participant was not scanned due to claustrophobia while in the scanner. After our analysis and performing quality assurance (see below for details), a further 8 participants were removed, leaving 18 final participants, between the ages of 21.3 and 53.4 years (mean age ± sd: 29.6 ± 8.7 years; 8 males). Sex, handedness, and date of birth were recorded on the day of the scan.

### 2.2 Ethics statement

Written informed consent was obtained from all participants. Ethics approval was granted by the Clinical Research Ethics Board at the University of British Columbia and BC Children’s & Women’s Hospital (H21-02686).

### 2.3 Scanning procedure

**Fig. 1.**
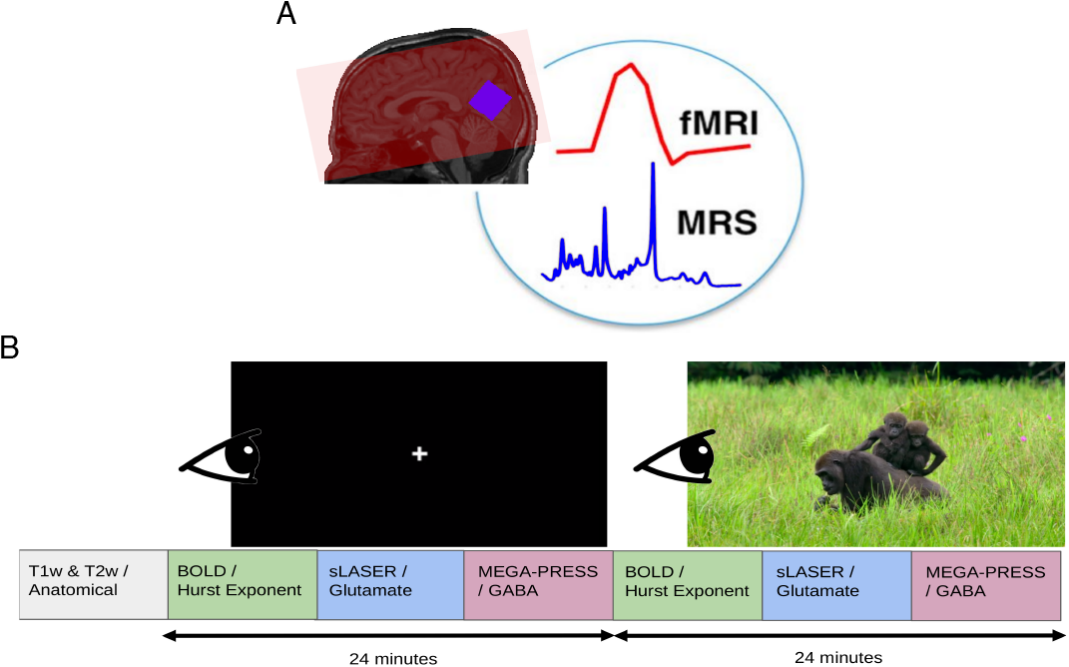
Overview of the MRI acquisition protocol. (A) fMRI coverage was across the whole brain (example coverage in red). MRS voxels were placed in the visual cortex (blue). Background image is of a T1w acquisition from a sample subject. Figure was inspired by Ip et al. (2017). (B) fMRI, sLASER, and MEGA-PRESS were acquired first with participants looking at a white cross for 24 minutes. Next, the same sequences were acquired with the participants viewing a nature documentary for an identical period of time.

After two anatomical sequences were acquired, participants were instructed to visually fixate on a cross-hair for 24 minutes (see Table 1 and below for more details). During this period, an fMRI, single-voxel semi Localization by Adiabatic SElective Refocusing (sLASER) (Oz & Tkáč, 2011), and single-voxel MEscher-Garwood Point-REsolved SpectroScopy (MEGA-PRESS) (Mescher et al., 1998) sequences were acquired (see Fig. 1A and Section 2.4). Next, participants were instructed to watch a nature documentary (Our Planet (2019), Episode 3, “Jungles” (Cordey, 2019)) for 24 minutes. During this period, another set of fMRI, sLASER, and MEGA-PRESS sequences were acquired. See Figure 1B for a visual representation of the scanning protocol. Total scan duration was approximately 1 hour of uninterrupted scanning (i.e., subjects were not removed from the scanner). All participants followed the same order of conditions: rest then movie watching; all participants saw the same movie segment, beginning at the same time during the scan.

**Table 1.** Summary of MRI acquisition details.

Sequence	TE (ms)	TR (ms)	Flip Angle	FOV	Slice Thickness (mm)	In-Plane Resolution (mm²)	Other Parameters	Time (mins)
3D T1-weighted FSPGR	2.176	7.216	12	256 × 256	0.9	0.9375 × 0.9375	−	5
3D T2-weighted CUBE	75.242	2,504.0	90	256 x 256	0.9	0.9375 × 0.9375	−	4
2D EPI Multi-Echo fMRI	12.2, 35.352, 58.504	1,500	52	64 x 64	3.6	3.5938 × 3.5938	Acceleration factor = 6	12
sLASER	35	2,000	90	−	28.0	28.0 x 28.0	Transients = 128*; spectral width = 5,000 Hz; data points = 4,096	4
MEGA-PRESS	68	1,800	90	−	28.0	28.0 x 28.0	Transients = 128 ON, 128 OFF; Spectral width = 5,000 Hz; data points = 4,096	8

* Note, the GE semi-LASER sequence averaged the 128 transients into groups of 4, each with 32 transients, before being saved as raw P-file.

TE = echo time; TR = repetition time; FOV = field of view; FSPGD = fast spoiled gradient echo; CUBE = a GE acronym; EPI = echo-planar imaging; fMRI = functional magnetic resonance imaging; sLASER = semi-localization by adiabatic selective refocusing; MEGA-PRESS = Mescher–Garwood point-resolved spectroscopy.

### 2.4 Acquisition details

Scans were performed at BC Children’s Hospital MRI Research Facility on a 3.0 Tesla GE Discovery MR750 scanner (scanner software version: DV26.0_R03) with a Nova Medical 32 channel head coil. Participants changed into scrubs and were screened by an MRI technologist. Participants were given wax earplugs and a fiducial was placed on their left temple. Participants were provided with an audio headset and blanket once lying down on the scanner bed. Since visual stimuli were rear-projected, position and angle of mirror above patient eyes were adjusted for optimal movie viewing.

The following MRI scans were acquired. A 3D T1-weighted sagittal fast spoiled gradient echo (FSPGR) sequence; a 3D T2-weighted sagittal CUBE; a 2D echo-planar imaging (EPI) multi-echo gradient-echo fMRI sequence; a single-voxel MEGA-PRESS sequence with CHESS (CHEmical Shift Selective saturation) water suppression; and a single-voxel sLASER sequence with VAPOR (VAriable Power and Optimized Relaxations) water suppression. Details are listed in Table 1.

For both MRS sequences, the voxel size was set to 2.8 x 2.8 x 2.8 cm^3^. MRS voxels were rotated and placed in the occipital lobe, aligned along the calcarine fissure, using the high-resolution T1w sequence as anatomical guide. Voxel placement was set once at the beginning and copied for the remaining MRS sequences. Glutamate was quantified from semi-LASER acquisitions as it offers the highest quality spectra for unedited metabolites (Wilson et al., 2019). The GE semi-LASER sequence automatically aligned and averaged every 32 transients, resulting in 4 averaged transients to process. GABA was measured using MEGA-PRESS, which applies frequency-selective editing pulses to isolate GABA from overlapping signals (Peek et al., 2023). This dual-sequence approach is standard in the field and reflects the differing spectral requirements of each metabolite (Van Veenendaal et al., 2018). Finally, blip-up and blip-down spin-echo versions of the fMRI sequence were acquired at the end to estimate the B0 non-uniformity map for fMRI phase distortion correction.

### 2.5 Image processing

Our full image processing pipeline has been accessed from our Github account page: github.com/WeberLab/EI_Hurst_Analysis

Images were downloaded offline from the scanner in raw Digital Imaging and Communications in Medicine (DICOM) format. DICOM files were then converted to Neuroimaging Informatics Technology Initiative (NIfTI) using Chris Rorden’s dcm2niix (Li et al., 2016) (v1.0.20211006) and then to Brain Imaging Data Structure (BIDS) (Gorgolewski, Auer, et al., 2016) format using dcm2bids (Boré et al., 2023) (v2.1.6). MRS files were downloaded as raw P files.

#### 2.5.1 Structural images

The T1w image was corrected for intensity non-uniformity with N4BiasFieldCorrection (Tustison et al., 2010) (ANTs (Avants et al., 2008); v2.3.335) to be used as a T1w-reference for the rest of the workflow. The T1w-reference was skull stripped using a Nipype (Gorgolewski, Esteban, et al., 2016) implementation of antsBrainExtraction.sh from ANTs; OASIS30ANTs was used as a target template. Fast (Zhang et al., 2001) (FSL (Smith et al., 2004) v.6.0.5.1:57b01774, RRID: SCR_002823) was used for brain tissue segmentation into cerebrospinal fluid (CSF), white matter (WM), and gray matter (GM). Brain surfaces were reconstructed with recon-all (Dale et al., 1999) (FreeSurfer (Dale et al., 1999) 7.3.2, RRID: SCR_001847). The previously estimated brain mask was refined with Mindboggle (Klein et al., 2017) (RRID:SCR_002438) to reconcile ANTs-derived and FreeSurfer-derived segmentations of cortical GM. AntsRegistration (Avants et al., 2008) (ANTs 2.3.3) was used to perform volume-based spatial normalization to two standard spaces: MNI152NLin2009cAsym and MNI152NLin6Asym. Normalization used brain-extracted versions of both T1w reference and T1w template.

#### 2.5.2 fMRI

Using fMRIPrep (Esteban et al., 2019), the shortest echo of the BOLD run was used to generate a reference volume (both skull stripped and skull included). Head-motion parameters with respect to the BOLD reference (transformation matrices as well as six corresponding rotation and translation parameters) were estimated before spatiotemporal filtering using mcflirt (Jenkinson et al., 2002) (FSL v6.0.5.1:57b01774). The fieldmap was aligned with rigid registration to the target EPI reference run. Field coefficients were mapped to the reference EPI using the transform. BOLD runs were slice-time corrected to 643 ms (half of slice acquisition range of 0–1,290 ms) using 3dTshift from AFNI (Cox, 1996) (RRIS: SCR_005927). To estimate T2* map from preprocessed EPI echoes, a voxel-wise fitting was performed by fitting the maximum number of echoes with reliable echoes in a particular voxel to a monoexponential signal decay model with nonlinear regression. Initial values were T2*/S0 estimates from a log-linear regression fit. This calculated T2* map was then used to optimally combine preprocessed BOLD across echoes using the method by Posse et al. (1999). The generated BOLD reference was then co-registered (six degrees of freedom) to the T1w reference with bbregister (FreeSurfer (Dale et al., 1999)) using boundary-based registration. First, a reference volume and its skull-stripped equivalent were generated with fMRIPrep. Confounding time series were calculated from preprocessed BOLD: framewise displacement (FD), DVARS, and three region-wise global signals. Tedana (DuPre et al., 2021) was then used to denoise the data by decomposing the multi-echo BOLD data via principal component analysis (PCA) and independent component analysis (ICA). The resulting components are automatically analyzed to determine whether they are TE dependent or TE independent. TE-dependent components were classified as BOLD, while TE-independent components were classified as non-BOLD and were discarded as part of data cleaning.

Participants were excluded from further analysis if their mean FD was > 0.15 mm.

### 2.6 MRS

sLASER data were processed using Osprey (Oeltzschner et al., 2020) (v2.9.5) and fit via the embedded LCModel (v6.3-1N) wrapper (Provencher, 2001). Because of substantial overlap among Glu and Gln resonances (Pasanta et al., 2023; Ramadan et al., 2013), in order to avoid errors in spectral assignment—especially since it is controversial whether Glu can reliably be separated from Gln at 3T (Zöllner et al., 2021; Zöllner et al., 2022)—we report Glx (Glu+ Glu) as the primary outcome measure. Individual Glu estimates are provided in Supplementary Materials (Table S5), alongside all other quantified metabolites. Several Osprey functions were modified to accommodate scanner-specific features of the GE implementation, where individual transients had been averaged by the scanner into four sub-spectra prior to export. These changes primarily affected GEload.m to correct water-scaling inconsistencies. RF coil combination was performed using generalized least squares (GLS) weighting (An et al., 2013; Bouchard & Mikkelsen, 2025).

MEGA-PRESS data were processed with Osprey, and the difference of the ON and OFF sequences was fit the LCModel algorithm. Due to the J-editing sequence of MEGA-PRESS, a challenge of GABA quantification is macromolecule quantification (Harris et al., 2015). As a result, we report GABA+, a measure which co-reports GABA with macromolecules. Macromolecules (MM) are expected to account for approximately 45% of the GABA+ signal (Harris et al., 2015). While a macromolecule-suppressed estimate of GABA seems ideal, a recent 25-site and multi-vendor study conducted at 3T found that GABA+ showed much lower coefficient of variation than MM-suppressed GABA, meaning that GABA+ is more consistent across research sites and MRI vendors (i.e., Philips, GE, Siemens) (Mikkelsen et al., 2019). Moreover, GABA+ shows greater reliability for both creatine-referenced and water-suppressed estimates (Mikkelsen et al., 2017; Mikkelsen et al., 2019). MM-suppressed GABA and GABA+ estimates are also correlated, albeit weakly to moderately so (Harris et al., 2015; Mikkelsen et al., 2017; Mikkelsen et al., 2019). Consequently, we report GABA+ to allow for easier comparison of our results with other studies as well as reproducibility. All metabolite values, including GABA, are reported in the Supplementary Materials (Table S6).

MRS voxels were co-registered to the T1w reference image and segmented by SPM12 (Friston et al., 2007) into CSF, GM, and WM. sLASER and MEGA-PRESS data were water scaled as well as tissue corrected and relaxation corrected by the Gasparovic et al. (2006) method. Concentrations are reported in millimoles. Full-width half-maximum (FWHM) of the single-Lorentzian fit of the water peak, signal-to-noise (SNR) ratio of the creatine signal, frequency shift, and Cramer–Rao lower bounds (CRLB) were calculated for quality assurance purposes. Osprey basis sets were used for linear combination. Metabolites included for both basis sets were Asc, Asp, Cr, CrCH2, EA, GABA, GPC, GSH, Gln, Glu, Gly, H2O, Lac, NAA, NAAG, PCh, PCr, PE, Ser, Tau, mI, and sI. Excitatory–inhibitory ratio (E:I) was calculated as [Glx from sLASER]/[GABA+ from MEGA-PRESS], a common practice to report E:I using MRS (Rideaux, 2021).

Participants were excluded from further analysis if any of their MRS scans had a water peak FWHM > 10 (Juchem et al., 2021).

To quantify the inter-subject consistency across the MRS masks, we computed a consensus mask using majority voting and calculated the mean Dice coefficient between each individual mask and the consensus.

#### 2.6.1 Re-analysis using Gannet

Glutamate and GABA were acquired using different MRS sequences and in separate blocks during the same scan session. While acquisitions were closely timed and performed under similar conditions, we cannot fully exclude the possibility of session-related variability affecting E:I ratio estimates. Therefore, we re-ran our analysis using the Glx and GABA+ values from the MEGA-PRESS sequence alone using Gannet (Edden et al., 2014) (v3.3.0).

### 2.7 Hurst exponent calculation

Hurst exponent was calculated from the power spectrum density (PSD) of the BOLD signal. A log–log plot was used, where log power was plotted against log frequency; generally, if a log–log plot results in a linear relationship, it is assumed that the mean slope of this line represents the power-law exponent (Zimmern, 2020). A PSD shows the distribution of signal variance (“power”) across frequencies. Complex signals are classified into two categories: fractional Gaussian noise (fGn) and fractional Brownian motion (fBm) (Duff et al., 2008; Eke et al., 2002). The former is a stationary signal (i.e., does not vary over time), while the latter is non-stationary with stationary increments (Eke et al., 2002). Most physiological signals consist of fBm, but fMRI BOLD is typically conceptualized as fGn once motion corrected; otherwise put, unprocessed BOLD signal is initially fBm which is converted to fGn with appropriate processing (E. Bullmore et al., 2004). fBm and fGn require distinct H calculation methods (Eke et al., 2002). PSD was estimated using Welch’s method (Welch, 1967) from the Python Scipy.Signal library (Virtanen et al., 2020). Data were divided into 8 windows of 50% overlap and averaged within each window, as is standard (Rubin et al., 2013). Welch’s method was selected based on prior comparisons with other Hurst exponent algorithms, which found it to perform best across all evaluation criteria (Rubin et al., 2013). The spectral index, β, was calculated from the full frequency spectrum. The spectral index was then converted to H using the following equation (Eke et al., 2002; Schaefer et al., 2014):

$$H=\frac{1+\beta }{2}.$$

Since it cannot be assumed that all fBm is removed from the signal, we employed the “extended Hurst” (H’) concept in this study: for 0 < H < 1, the signal is understood as fGn, while for 1 < H < 2, the signal is understood to be fBm (O. Campbell et al., 2022; Eke et al., 2000; Hartmann et al., 2013). More generally, it is assumed that when 0.5 < H < 1.5, the signal displays 1/f behaviour (Zimmern, 2020). H was calculated for all voxels in the brain of each subject. A brain mask was then applied which included only GM and the region of the MRS voxel in the visual cortex. H was averaged across the brain mask area, using only non-zero voxels.

### 2.8 Statistics

All statistical analyses were performed using R (Team, 2021) (v4.5.1) and RStudio.

A linear mixed-effect model was used to test for the correlation of H and E:I, with H as the dependent variable, Glx/GABA+ as the primary predictor of interest, and Condition (rest vs. movie watching) as a fixed effect. Mean FD, FWHM (sLASER), FWHM (MEGA-PRESS), Frequency Shift (sLASER), and Frequency Shift (MEGA-PRESS) were included as covariates, and subjects were modelled as random effects:

$$\begin{array}{ccc} \text{H}∼ & \frac{\text{Glx}}{\text{GABA+}}+\text{Condition}+\text{meanFD} & \\ & +{\text{FWHM}}_{\text{sLASER}}+{\text{FWHM}}_{\text{MEGA-}PRESS} & \\ & +{\text{FreqShift}}_{\text{sLASER}}+{\text{FreqShift}}_{\text{MEGA-}PRESS} & \\ & +\left(1\text{|Subject}\right). & \end{array}$$

A post hoc calculation of the power of the model was calculated using powerSim from the simr package using 1,000 simulations.

Exploratory post hoc analyses were then performed. Difference in the means of H, Glx, and GABA+ between rest and movie conditions was assessed using paired Student’s t-tests (Student, 1908). Correlations between H and Glx, GABA+, and E:I were calculated using Pearson’s method (Freedman et al., 2007).

## 3 Results

### 3.1 Participant demographics

**Fig. 2.**
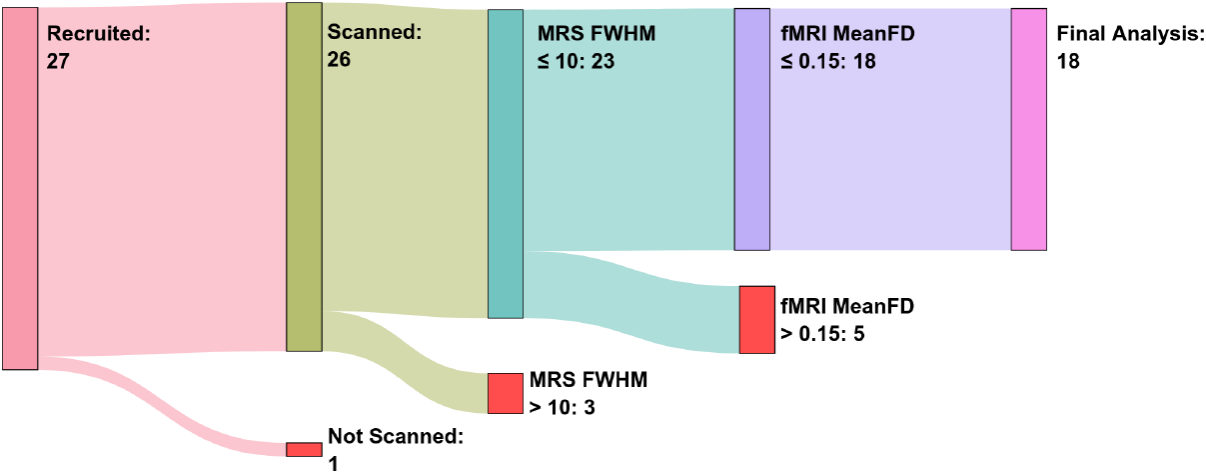
Flowchart of participant, recruitment, scanning, and exclusion. The number of participants at each stage is indicated within each node. Participants were excluded based on MRS FWHM and fMRI Mean FD thresholds, resulting in 18 participants included in the final analysis.

Twenty-seven participants were originally recruited for the study. Twenty-six of these participants were successfully scanned, but one participant experienced claustrophobia and chose not to continue. Of the remaining 26 participants, 18 were included in the final analysis: 3 were removed due to low MRS quality (FWHM > 10) and 5 were removed due to low fMRI quality (mean FD > 0.15 mm) (see Fig. 2).

The final study sample included 8 males and 10 females between ages 21.3 and 53.4 years, with a mean age and standard deviation of 29.6 ± 8.7 years.

### 3.2 Data summary

**Fig. 3.**
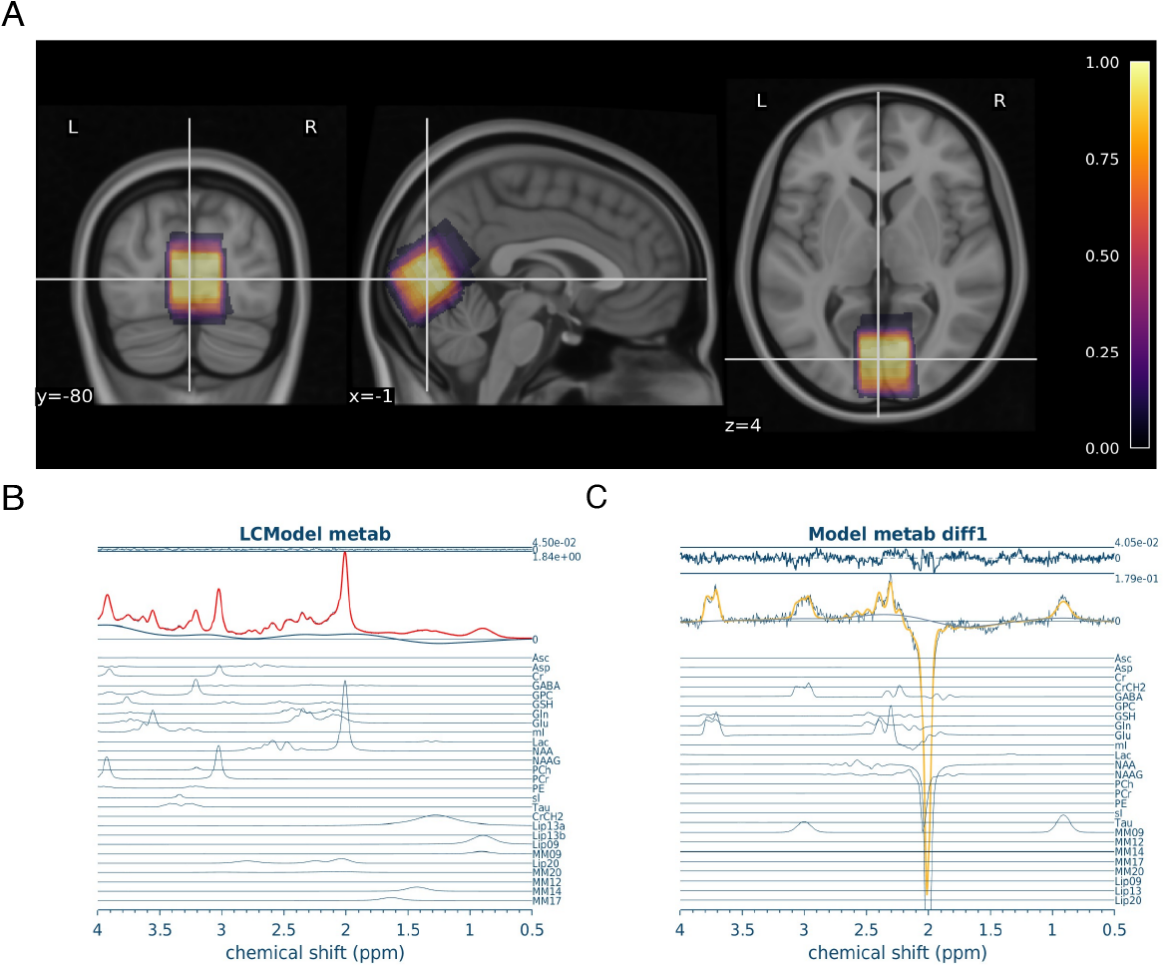
MRS data quality. (A) Average MRS location. Coloured overlay of the average voxel location across all 19 participants, from 0 (black) to 1 (white). Brighter yellow represents voxel locations shared by all participants, while darker purple represents voxel locations unique to participants. MRS voxels were registered to MNI space and averaged. Underlay is T1w MNI152 at 0.5 mm resolution. (B) Sample Osprey sLASER Spectrum. Red spectrum indicates overall model fit. Model fits for individual metabolites are shown in blue below overall fit. (C) Sample Osprey MEGA-PRESS Spectrum. Yellow spectrum indicates overall model fit. Model fits for individual metabolites are shown in blue below overall fit.

An average of all MRS voxel placements are shown in Figure 3A, and a sample of the Osprey sLASER and MEGA-PRESS spectrum fits at rest is shown in Figure 3B and C, respectively. The mean consensus-based inter-subject Dice score for MRS voxel placements was 0.77 ± 0.14 (range: [0.35, 0.95]).

Average water FWHM, creatine SNR, and frequency shift are reported in Supplementary Materials (Table S1).

Average voxel rotation along the x-axis, along with average tissue fractions is reported in Supplementary Materials (Table S2).

**Fig. 4.**
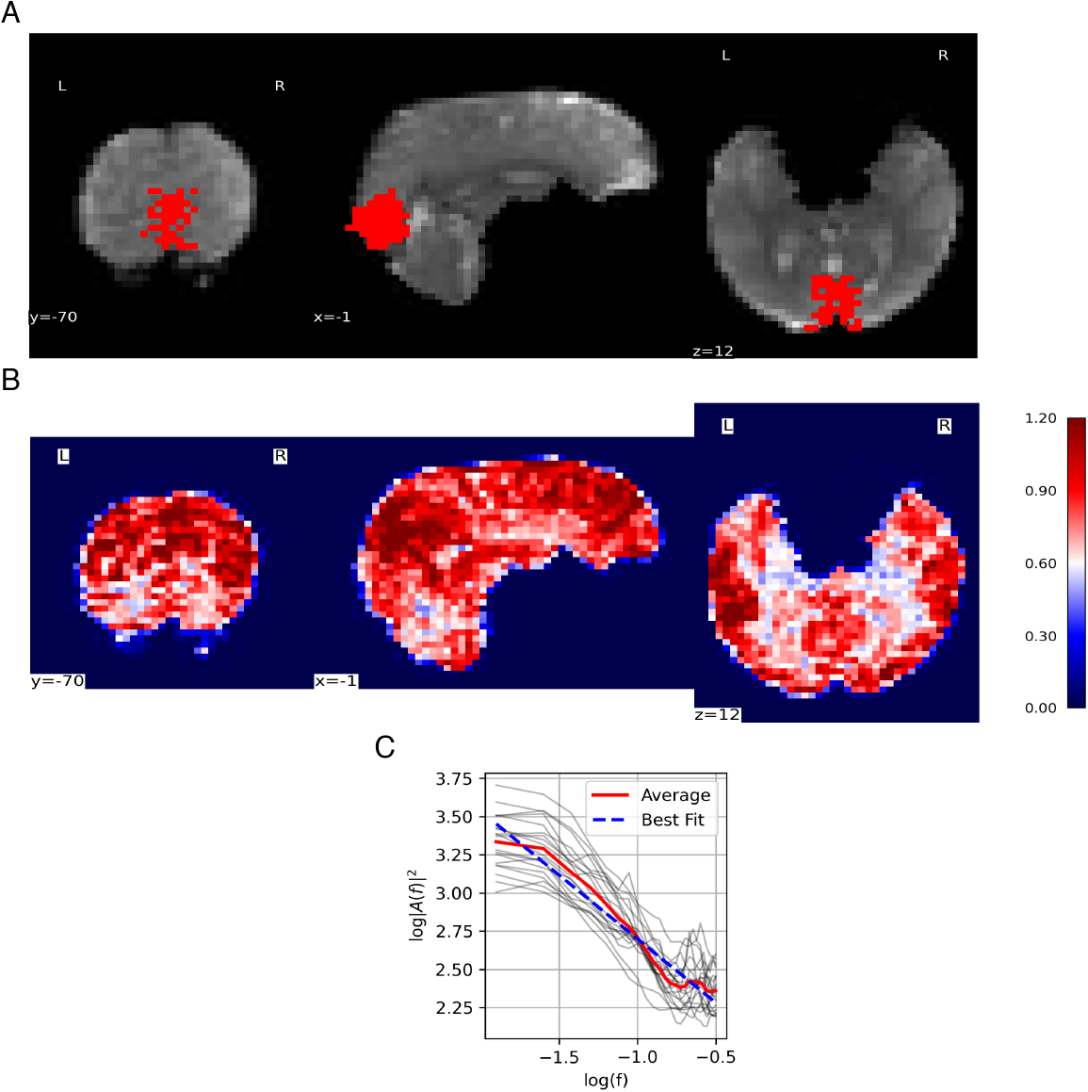
fMRI data quality. (A) Sample fMRI grey matter mask within the MRS voxel. Background: a sample coronal, sagittal, and axial slice is displayed of the mean fMRI scan from the rest acquisition. Foreground: the grey matter/MRS mask used to calculate mean H. (B) Sample H map for whole brain. Coronal, sagittal, and axial views of are shown, colour coded by H values. Colour coding shows evident tissue differentiation by H value (i.e., grey matter (red), white matter (light red), and cerebrospinal fluid (white)). (C) Sample PSD spectrum. All participants’ PSD spectrums during rest are plotted using light grey lines. Mean PSD is plotted in solid red. Mean linear regression line is plotted in dashed blue. H for each participant was calculated from the slope of the mean linear regression line.

A sample of the combined grey matter and MRS voxel mask used to average H values, along with a sample Hurst exponent map, and sample fits for H calculation during rest is shown in Figure 4A, B, and C, respectively.

**Fig. 5.**
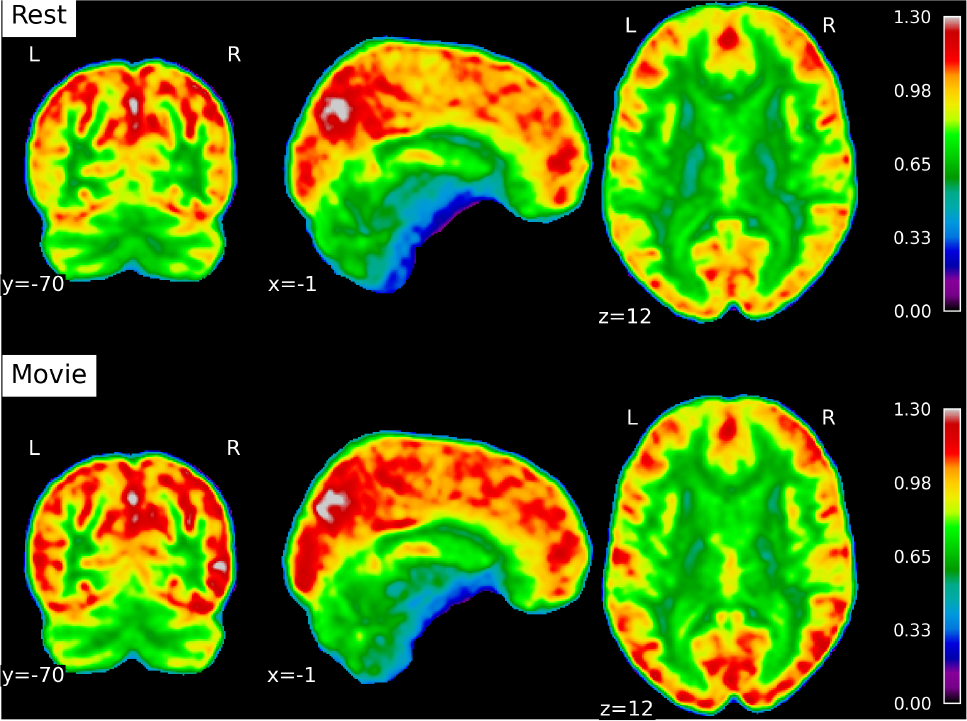
Average Hurst maps for rest and movie conditions. Group-averaged maps of the Hurst exponent computed to visualize whole-brain patterns across sessions: rest (top) and movie watching (bottom).

Figure 5 shows the average Hurst maps during rest and movie watching.

### 3.3 Linear-mixed effects model: H and E:I

To examine the relationship between H and E:I, we ran a linear mixed-effects model. The model’s total explanatory power was substantial (conditional r^2^ = 0.79), with fixed effects (marginal r^2^) equal to 0.22. The model’s intercept, corresponding to EI = 0, Condition = Movie, FWHM_sLASER_ = 0, FWHM_MEGA-PRESS_ = 0, FreqShift_sLASER_ = 0, FreqShift_MEGA-PRESS_ = 0, and meanFD = 0, was 1.3 (95% CI [0.66, 1.95], t(26) = 3.95, p < 0.01).

Among the fixed effects, only the effect of Condition [Rest] (beta = -0.07, 95% CI [-0.11, -0.02], t(26) = -2.84, p = 0.02); and Frequency Shift (sLASER) (beta = 0.03, 95% CI [0.01, 0.06], t(26) = 2.31, p = 0.05) was statistically significant. All other predictors—including E:I (beta = 0.07, 95% CI [-0.05, 0.19], t(26) = 1.12, p = 0.28); along with FWHM_MEGA-PRESS_ (beta = 0.01, 95% CI [-0.05, 0.07], t(26) = 0.28, p = 0.78); FWHM_sLASER_ (beta = -0.02, 95% CI [-0.09, 0.05], t(26) = -0.58, p = 0.57); FreqShift_MEGA-PRESS_ (beta = 0.03, 95% CI [-0.01, 0.06], t(26) = 1.56, p = 0.14); FreqShift_sLASER_ (beta = 0.03, 95% CI [0.01, 0.06], t(26) = 2.31, p = 0.05); and mean FD (beta = -0.59, 95% CI [-2.14, 0.96], t(26) = -0.75, p = 0.46)—were not statistically significant.

### 3.4 Post hoc analyses

Diagnostics of our linear-mixed effects model is given in the Supplementary Materials (Fig. S1; Tables S3 and S4).

To calculate the post hoc power of our model, we required an effect size. While *in silico* and animal studies indicate that changes in E:I ratio influence the Hurst exponent (Trakoshis et al., 2020), these models do not provide standardized effect sizes, and translation to human neuroimaging remains uncertain. Therefore, we used several effect sizes, and report here the power of each. The effect sizes we used to calculate power were the value calculated from our model (beta = 0.07); and beta values two and three times that size: 0.14 and 0.21. As a reminder, beta represents the increase of H by that amount per every increase of E:I by 1. The power for these effect sizes was 25.7%, 64.2%, and 93%, respectively.

Furthermore, as our spectroscopy quality metrics could be highly correlated with each other (e.g., FWHM measures from the two sequences, or frequency shifts), we checked variance inflation factors (VIFs) to ensure all values were < 5 (generally acceptable). VIFs were EI = 1.24, Condition = 1.55, FWHMsLASER = 1.76, FWHMMEGA = 1.45, FreqShiftsLASER = 1.15, FreqShiftMEGA = 1.48, and MeanFD = 1.28.

**Fig. 6.**
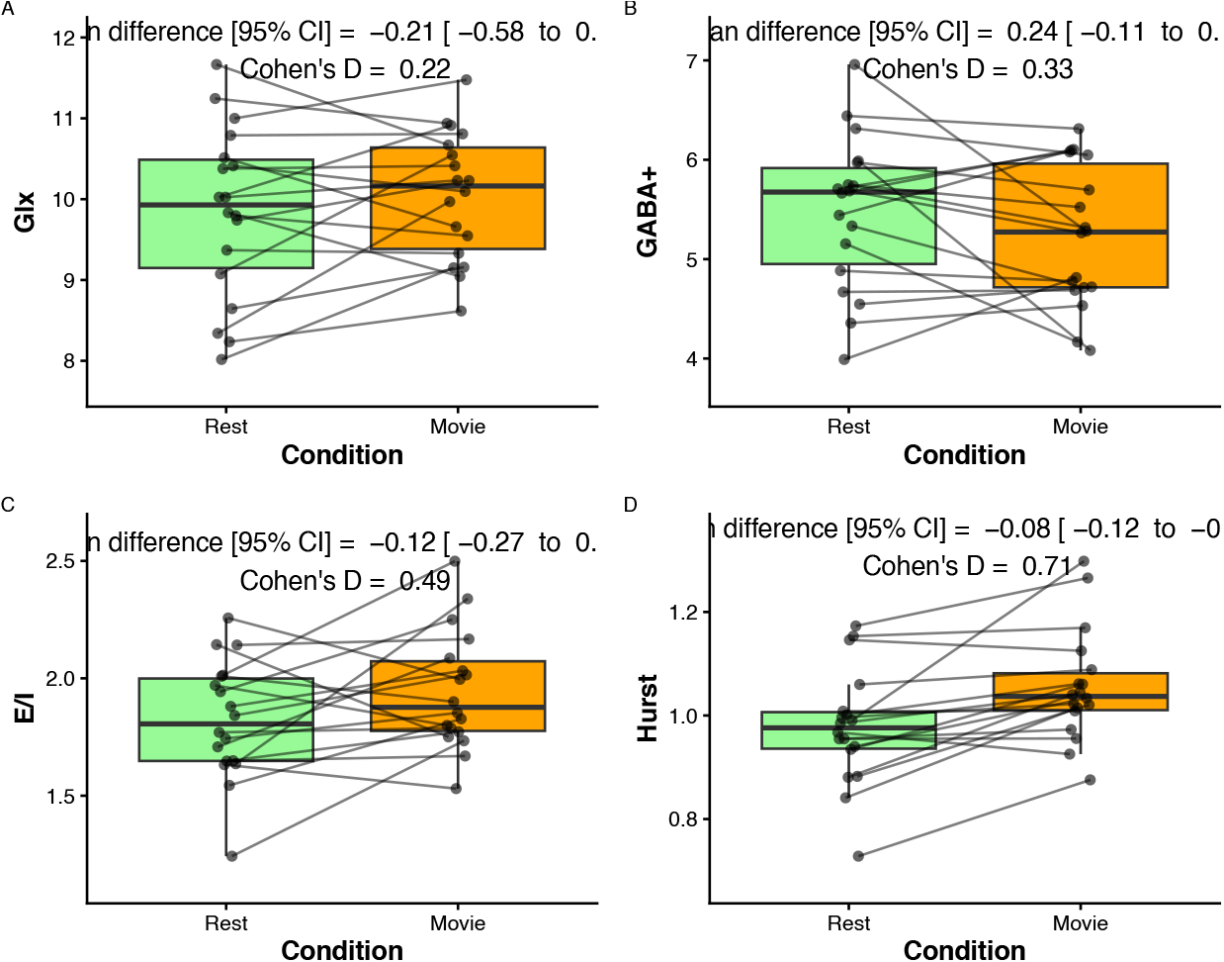
Paired comparison of metabolite values during rest (green) and movie-watching (orange) conditions. (A) Glx (mM), (B) GABA+ (mM), and (C) E:I. Paired dots represent the same participant across conditions. Mean difference with 95% confidence intervals and as Cohen’s D are reported at the top of each plot.

Mean ± sd of the main metabolites, their Cramer–Rao lower bounds, E:I, and H during rest and movie watching are reported in Table 2. Boxplots between rest and movie watching are shown in Figure 6. Neither Glx nor GABA+ was different between movie watching and rest conditions. E:I ratio did not change between conditions either. H was found to be greater during movie watching than rest.

**Table 2.** Summary of main measures.

	Rest	Movie	p-value
Glx	9.84 ± 1.06	10.04 ± 0.79	0.26
GABA+	5.48 ± 0.77	5.23 ± 0.71	0.17
E:I Ratio	1.82 ± 0.25	1.94 ± 0.25	0.08
H	0.98 ± 0.98	1.05 ± 1.05	<0.01

Glx and GABA+ values are in millimoles.

**Fig. 7.**
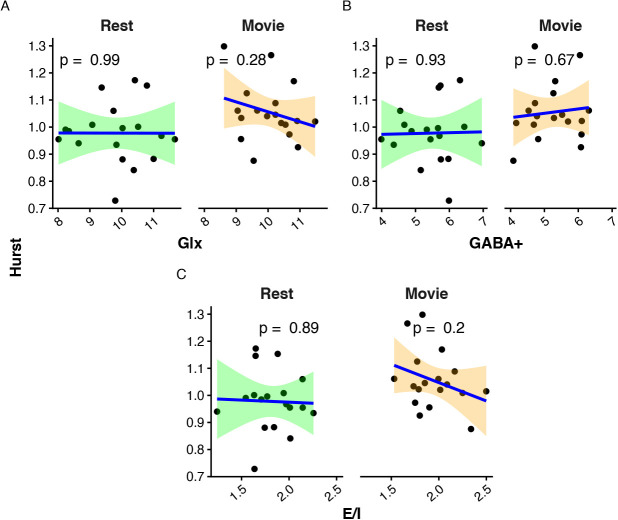
Scatter plots of H vs. metabolites. (A) Glx (mM); (B) GABA+ (mM); and (C) E:I. Rest is in green, while movie watching is in orange. p-Values are reported at the top of each plot.

H was not found to correlate with Glx, GABA+, or E:I, during rest or movie watching (Fig. 7).

#### 3.4.1 Data quality

Mean FD was not correlated with H during rest (r = -0.33, p = 0.18) but was moderately negatively correlated with H during movie watching (r = -0.49, p = 0.04; see Supplementary Materials, Fig. S2A).

Glx and GABA+ were tested for associations with FWHM values during rest and movie watching. No significant correlations were found (see Supplementary Materials, Fig. S2B and C). Glx and GABA+ were tested for associations with frequency shift during rest and movie watching. No significant correlations were found (see Supplementary Materials, Fig. S2D and E).

Water FWHM and creatine SNR (CR-SNR) for both sLASER and MEGA-PRESS were analyzed pre- vs. post-fMRI. Supplementary Tables S7, S8, and S9 present the paired t-tests and subject-level rest/movie values, while Supplementary Figure S3 shows the individual trajectories. No systematic drifts were detected (all p > 0.2 for FWHM; p > 0.3 for SNR). Furthermore, a group-averaged plot of the frequency shift between rest and movie watching is given in the Supplementary Materials (Fig. S4).

### 3.5 Re-analysis using Gannet

The re-analysis of our results using only the MEGA-PRESS sequence and Gannet did not result in any significant changes from the results reported above. We have included these results in the Supplementary Materials (Figs. S5–S8; Tables S10 and S11).

## 4 Discussion

This study provides the first *in vivo* human investigation of the relationship between H and the E:I ratio, measured using fMRI and MRS-derived glutamate and GABA levels, respectively. No association was observed between H and E:I, suggesting that these indices may reflect distinct dimensions of neural dynamics. During movie watching, H increased relative to rest, consistent with stronger long-range temporal dependencies in BOLD activity during visual stimulation. In contrast, Glx, GABA+, and their ratio (E:I) remained stable across conditions. These results indicate that functional signal complexity, as indexed by H, is sensitive to sensory engagement but does not appear to covary with MRS-derived estimates of excitatory–inhibitory balance under the current experimental conditions.

An increase in H during movie watching relative to rest aligns with previous work from our laboratory (O. Campbell et al., 2022), which reported increased H in the visual network (as defined by Yeo’s seven resting-state networks (Thomas Yeo et al., 2011)) during movie watching using data from the Human Connectome Project (Van Essen et al., 2012). While this increase in H is consistent with our prior findings, other studies have reported decreases in H during active tasks (Barnes et al., 2009; Churchill et al., 2016; Ciuciu et al., 2014; He, 2011). The current results, therefore, suggest that the naturalistic, passive nature of movie watching elicits a different effect on H compared with more active tasks. This interpretation is consistent with evidence indicating distinct neural responses and BOLD signal characteristics between conventional active visual tasks and naturalistic passive visual stimuli (O. Campbell et al., 2022; Hasson et al., 2010). Higher H during movie watching may reflect richer scaling properties that support the continuous integration of visual stimuli (O. Campbell et al., 2022).

No significant changes were observed in either Glx or GABA+ between conditions. Consequently, the E:I ratio remained stable. The experimental design included both resting and movie-watching conditions to induce measurable differences in H, Glx, and GABA+, providing a wider range of values to test for correlations between these metrics. However, the absence of condition-related differences in Glx and GABA+ complicates interpretation of the null findings. Establishing such changes would have provided a foundation for directly examining the correlation between H and the E:I ratio. Without clear evidence that the paradigm elicited detectable neurochemical variation, it remains uncertain whether the lack of association between H and E:I reflects a genuine independence of these measures or limited sensitivity of the MRS protocol to capture subtle metabolic changes.

A recent meta-analysis of studies measuring Glu and Glx (Pasanta et al., 2023) reported a minimal task-induced increase in Glu and Glx within the visual cortex, primarily in the context of pain, learning, or motor paradigms rather than visual stimulation. Additionally, many of the studies included were conducted at 7T, offering increased sensitivity for detecting changes in Glu and Glx. Nonetheless, several studies have demonstrated changes in Glu and Glx at 3T (Apšvalka et al., 2015; Cleve et al., 2017; Gutzeit et al., 2013). Regarding our GABA+ findings, the same meta-analysis (Pasanta et al., 2023) reported no task-dependent change in GABA within the visual cortex. This may be attributable to technical difficulties associated with capturing GABA levels using MRS at 3T due to its low concentration and spectral overlap with more abundant metabolites (Pasanta et al., 2023). Whereas several studies have successfully reported changes in GABA at 3T using different paradigms and/or regions of interest (Floyer-Lea et al., 2006; Sampaio-Baptista et al., 2015; Stagg et al., 2011).

Another reason changes in Glx or GABA+ may not have been observed could be due to our use of a block design: collecting 24 minutes of rest data, then collecting 24 minutes of movie-watching data. While a block design has the advantage of a more robust metabolite quantification due to greater signal averaging, brain homeostatis during these long blocks may lead to an erasure of any real metabolic changes (Apšvalka et al., 2015; Mangia et al., 2012). Indeed, Pasanta et al. (2023) and Archibald et al. (2020) reported larger metabolite changes during event-related paradigms than block designs, underscoring how task timing and structure can strongly influence the detectability of metabolic modulation with MRS.

The concept of E:I balance encompasses multiple biological scales and mechanisms beyond neurotransmitter concentrations, including the relative densities of excitatory and inhibitory neurons, synaptic properties, interneuron subtype interactions, and electrophysiological signatures such as 1/f spectral scaling. While the glutamate-to-GABA ratio measured via MRS provides a noninvasive—albeit indirect—estimate of neurochemical E:I balance in humans, it remains a simplification of the underlying complexity. MRS-derived values reflect total glutamate and GABA metabolite pools, predominantly intracellular and metabolically active, rather than direct synaptic neurotransmitter release underlying real-time excitation and inhibition. Moreover, conventional MRS measures such as Glx and GABA+ include contributions from glutamine and macromolecules, respectively, limiting their chemical specificity. Therefore, while these metabolites serve as useful proxies related to excitatory and inhibitory neural systems, caution is warranted when interpreting them as direct indices of neurotransmission. Nonetheless, these metabolic pools provide valuable insight into the overall balance of excitation and inhibition within the brain’s microenvironment and remain widely used in human *in vivo* studies of neural function and dysfunction. Finally, few studies have directly quantified both glutamate and GABA in the same individuals, highlighting the novelty and importance of this approach. Our study acknowledges these limitations and aims to provide a step towards linking neurochemical measures with large-scale brain dynamics related to criticality.

Alternatively, the present findings may in fact indicate that H and E:I are not directly coupled, or that their relationship is more complex than a simple linear association. This would perhaps not be surprising given the large disparity of findings in the literature, especially with regard to the directionality and linearity of the proposed E:I–Hurst relationship (Baumgarten & Bornholdt, 2019; Bruining et al., 2020; Gao et al., 2017; Liang et al., 2025; Lombardi et al., 2017; Poil et al., 2012; Trakoshis et al., 2020) (see Table 3). The heterogeneity across these studies underscores the challenges of studying this phenomenon and suggests that any potential E:I–Hurst relationship may depend on experimental context, data acquisition methods, or analysis strategies. Further work is needed to clarify the true nature of this relationship and the conditions under which it might emerge.

**Table 3.** Summary of methods for existing E:I–Hurst studies.

Citation	Study Type	H Data Type	H Calculation Method	E:I Type	E:I–Hurst Relationship
Poil et al. (2012)	Computational with in-house simulated model	Neuronal avalanche size	Detrended fluctuation analysis (DFA)	Structural: number of E-to-I neurons	Inverse U
Bruining et al. (2020)	Computational with model by Poil et al. (2012); modified in-house	Neuronal oscillation amplitude	DFA	Structural: number of E-to-I synapses	Inverse U
Gao et al. (2017)	Computational; in vivo in rats and macaques	Local field potential (LFP)	PSD	Estimated from LFP	Positive linear
Lombardi et al. (2017)	Computational with in-house model	Neuronal avalanche size	PSD	Structural: number of E-to-I neurons	Negative linear
Trakoshis et al. (2020)	Computational with simulated data; in vivo in mice	fMRI BOLD signal	Wavelet-based maximum likelihood method	E-to-I synaptic conductance	Negative linear

Our findings specifically show an absence of a robust association between the Hurst exponent and MRS-derived E:I ratio, challenging the assumption that these measures directly reflect a shared underlying mechanism, such as the brain’s distance from criticality. One possibility is that while both H and E:I may independently vary with neural excitability, their relationship is nonlinear or not detectable at the temporal and spatial scales of fMRI and MRS. Additionally, the E:I balance estimated via glutamate and GABA concentrations may reflect metabolic rather than synaptic activity, limiting its correspondence with network dynamics captured by BOLD signal autocorrelation. These results have important implications for studies that use H as a proxy for E:I, particularly in neurodevelopmental or neurodegenerative contexts, and suggest caution in interpreting H as a stand-in for neurochemical balance without direct validation. Future research should investigate whether the relationship emerges under task-driven conditions, in specific brain regions, or in populations with pronounced E:I dysregulation.

Finally, it is also possible that while an E:I–Hurst relationship exists, it is not observed within the visual cortex. This theory seems plausible given that MRS studies of disrupted E:I, mostly conducted within the context of autistic adults, have found changes in E:I within other brain regions such as the anterior cingulate cortex, frontal lobe, or temporal lobe (Ajram et al., 2019). Moreover, findings with reference to changes in excitatory or inhibitory neurotransmitters within the visual cortex tend to be difficult to capture, perhaps indicating that E:I shows less changes in this region (Pasanta et al., 2023). However, this would suggest that the E:I–H relationship would be region dependent, and, therefore, not a global theory as it is often portrayed.

As both H and E:I have been known to change with age, we did not seek to limit our sample to any specific age range. Furthermore, other physiological details—such as caffeine, nicotine, phase of the menstrual cycle, gender, and medication use—were not collected, as the core E:I–Hurst theory posits a global relationship that should hold irrespective of specific brain states or transient physiological factors. Additionally, our within-subjects design strengthens this approach: our core analyses assessing changes between resting state and movie watching were paired, with each participant serving as their own control. This reduces the influence of inter-individual variability, including age-related and other brain-state effects.

### 4.1 Limitations and strengths

Beyond the limitations already mentioned (field strength, visual cortex, passive task, block design), another limitation was our small sample size. As this was a pilot study, 26 participants were initially scanned. Once individuals were excluded for poor MRI quality, only 18 participants’ data were analyzed. With this small sample size, it is difficult to make conclusions about a concept as complex as the E:I–Hurst relationship.

It is also important to note that MRS measures reflect total glutamate and GABA metabolite pools, predominantly intracellular and involved in metabolic processes, rather than direct synaptic neurotransmitter release. Furthermore, commonly used measures such as Glx and GABA+ include signals from glutamine and macromolecules, respectively, limiting their chemical specificity. Therefore, while these metabolites serve as useful proxies related to excitatory and inhibitory neural systems, caution is warranted when interpreting them as direct indices of neurotransmission. Nonetheless, these metabolic pools provide valuable insight into the overall balance of excitation and inhibition within the brain’s microenvironment and remain widely used in human *in vivo* studies of neural function and dysfunction. Although voxel placement was guided by anatomical landmarks and performed by trained personnel, some variability in voxel location across subjects and sessions may have introduced noise in metabolite quantification. Future studies may benefit from automated alignment and formal voxel overlap analysis to reduce this source of variability. Furthermore, our MRS voxels were placed once for the first MRS sequence, and copied for the subsequent MRS runs. This “cloning” assumes nominal stability and does not capture any potential effective sampling differences due to head motion or shim-related changes. Future studies may also wish to acquire high-resolution anatomical scans before each MRS run in order to re-align the MRS voxel and to quantify intra-subject voxel movement; voxel dice coefficient: overlap of runs (ideally >0.90); center-of-mass distance: Euclidean shift (mm) between runs (e.g., threshold >5 mm outlier). We did not explicitly monitor scanner frequency drift following the fMRI runs. Heating-related drift can affect the stability of MRS sequences, potentially introducing variability in glutamate and GABA quantification (Hui et al., 2021). While all MRS data were visually inspected for quality, we cannot rule out subtle effects of drift, which may have contributed to noise in the E:I measurements. We included frequency drift in our linear mixed-effects models in an attempt to control for these confounds. For future research, we highly recommend prospective drift corrections to address these issues. Finally, the order of scanning conditions (rest followed by movie watching) was not pseudorandomized. This may introduce potential order-related confounds, such as physiological adaptation or scanner drift, which could influence the observed differences between conditions.

An additional avenue for future research involves dynamic fMRS, which seeks to capture time-varying changes in metabolite levels during task performance. While promising, such approaches currently face substantial limitations in signal-to-noise ratio and temporal resolution, particularly for edited GABA measures. Our study was not optimized for dynamic fitting, but future work could explore how fluctuations in neurometabolites relate to moment-to-moment variations in BOLD signal complexity.

We hope that by future researchers can use our reported effect sizes to calculate potential sample sizes. We would also like to list some of the strengths of our study, which include using sLASER (as opposed to PRESS), which has been shown to have enhanced detection of complex multiplets such as Glx/Glu (Wilson et al., 2019); using the J-editing sequence MEGA-PRESS for improved GABA detection (Peek et al., 2023); using a large MRS voxel size (~22 ml) as per consensus recommendations (Choi et al., 2021; A. Lin et al., 2021; Peek et al., 2020); measuring H within the same region as our single-voxel MRS; and using multi-echo fMRI for improved motion artefact regression (Kundu et al., 2017).

### 4.2 Lessons for future researchers

Finally, we hope that we can provide some guidance to future researchers. The following is a non-exhaustive list of suggestions for future work:

- future studies should consider using ultra-high-field 7T MRI, which provides improved spectral resolution and more reliable detection of Glu and GABA compared with conventional 3T MRI (Terpstra et al., 2016);
- examining paradigms that have consistently demonstrated alterations in these metabolites, such as pain studies, may enhance the likelihood of detecting changes in Glu and GABA (Archibald et al., 2020; Cleve et al., 2017; Gutzeit et al., 2013);
- exploring brain regions beyond the visual cortex, such as the anterior cingulate cortex, which is commonly implicated in pain processing (Archibald et al., 2020; Gussew et al., 2010; Gutzeit et al., 2011; Mullins et al., 2005);
- including a more diverse participant sample—beyond healthy controls—may help capture a wider range of H and E:I values, potentially improving sensitivity to metabolite-related changes;
- increasing sample size in order to detect small effect sizes;
- using an event-related design, as opposed to the block design we used, may provide more sensitivity to detecting rapid and transient neural responses due to its ability to isolate specific events within the scan session (Mullins, 2018). However, see Pasanta et al. (2023) for a longer discussion and possible downsides to this approach;
- and finally, using a combined fMRI-MRS sequence (Dwyer et al., 2021; Ip et al., 2017) to measure BOLD and Glu/GABA near simultaneously.

Together, these considerations may help in overcoming the limitations observed in the present study and contribute to a clearer understanding of the potential relationship between E:I and H.

## 5 Conclusion

In conclusion, the results do not support a relationship between H and the E:I ratio in the visual cortex either during rest or during movie watching at 3T in humans. Although a task-related increase in H was observed, no accompanying changes were detected in Glu, GABA, or E:I between movie watching and rest. Comparing our findings with the broader literature, E:I balance may be too subtle to be detected with conventional 3T MRS methods (Terpstra et al., 2016). Therefore, higher-field (7T) or multi-voxel fMRS studies would be needed to confirm. With regard to the broader E:I–Hurst relationship, we similarly suggest that either this relationship is insufficiently captured with our methods, or that the relationship between these two variables may be more complex than originally envisaged—perhaps they are not directly related, but rather connected through other mediating variables in a non-linear fashion. To our knowledge, this is the first *in vivo* human study to test for this relationship. It is our hope that as the literature grows, more authors will examine this relationship with respect to other brain regions and using other methods, and will use the lessons learned in this study to inform their own. Hopefully then it will be possible to corroborate findings to probe the complex relationships that may exist with regard to H and E:I in the human brain.

## Supplementary Material

Supplementary Material

## Data Availability

All code used in this paper is available at github.com/WeberLab/EI_Hurst_Analysis. The raw MRI data used in this paper are available by contacting the author. The manuscript was written in a “reproducible manner”: the entire manuscript, including statistics reported, figures, and tables, is reproduced here: weberlab.github.io/EI_Hurst_Manuscript/.
