# Supplementary material for: Does the brain’s E:I balance really shape long-range temporal correlations? Lessons learned from 3T MRI
Source: Imaging Neurosci (Camb). 2026 May 29;4:IMAG.a.1252. doi: 10.1162/IMAG.a.1252 (PMC13224315; doi:10.1162/IMAG.a.1252)
Supplement: Supplementary Material [file IMAG.a.1252_supp.pdf]

Supplementary Material

MRS quality

A summary of MRS quality, such as water FWHM, Cr SNR, and frequency shift are provided in Table S1.

Measure	Rest sLASER	Rest MEGA-PRESS	Movie sLASER	Movie MEGA-PRESS
water FWHM (Hz)	8.47 ± 0.66	7.86 ± 0.8	8.32 ± 0.72	7.95 ± 0.82
Cr SNR	147.61 ± 20.46	101.05 ± 21.42	140.6 ± 26	98.75 ± 20.01
Frequency Shift (ppm)	-3.08 ± 0.81	-3.98 ± 0.94	-3.12 ± 0.76	-3.85 ± 1.05

*Table S1***. *Summary of spectroscopy quality measures.*** *FWHM = full-width half-maximum; Cr = creatine; SNR = signal-to-noise ratio.*

A summary of voxel placement statistics are provided in Table S2.

Measure	Amount
Voxel Rotation (degrees)	-39 ± 8.13
GM Tissue Fraction	0.64 ± 0.04
WM Tissue Fraction	0.24 ± 0.04
CSF Tissue Fraction	0.12 ± 0.03

*Table S2***. *Summary of spectroscopy voxel placement values*** *GM = grey matter; WM = white matter; CSF = cerebrospinal fluid.*

Linear-mixed effects model diagnostics

Linear-mixed effects model diagnostics are provided in Figure S1.

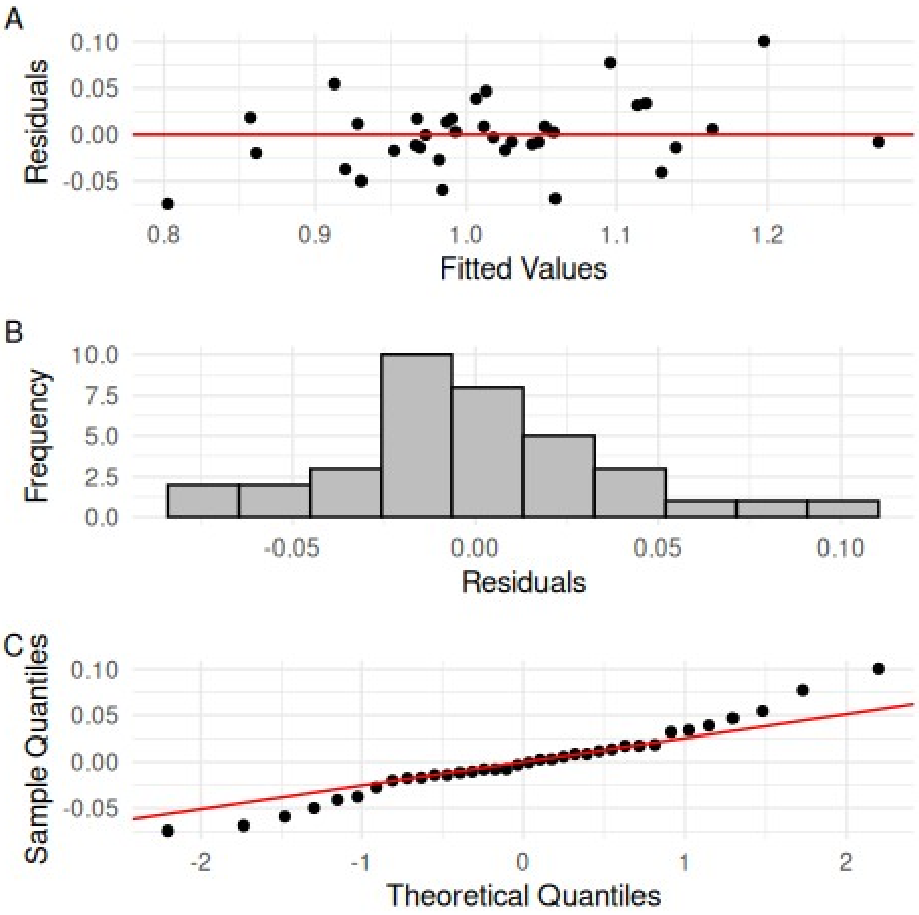

*Figure S1***. Linear-mixed effects model tests for normality.** *Model diagnostics for the linear mixed effects model predicting Hurst exponent from the EI ratio. A) Residuals vs. fitted values showing homoscedasticity. B) Histogram of residuals. C) Q-Q plot indicating normally distributed residuals.*

We report Spearman correlations between E:I and H within each condition (Table S3). Spearman rho values were modest and negative (Movie: ρ = -0.3, p = 0.23; Rest: ρ = -0.12, p = 0.62), and consistent with the primary analyses in direction but not statistically significant.

Condition	spearman_ei_hurst	p_value
Movie	-0.300	0.225
Rest	-0.125	0.621

*Table S3***.** *Spearman correlation between EI and Hurst by condition.*

We also refit the linear mixed-effects model after removing the top/bottom 10% of observations in E:I, in H, and in the intersection of both (Table S4). Across these trimmed datasets, the E:I fixed-effect estimate remained small and its 95% confidence interval continued to include zero, indicating that the primary conclusion is not driven by extreme values in either E:I or H. Trimming was performed on the pooled long-format dataset (rest and movie rows) to match the model specification.

Taken together with the non-parametric Spearman correlations (Table S3), these analyses indicate that the E:I–H association is weak in this pilot dataset and that our primary inference (a small effect with confidence intervals spanning zero) is unchanged under non-parametric estimation and reasonable exclusion of potential outliers.

Model	Estimate	SE	CI_low	CI_high
Full sample	0.068	0.061	-0.052	0.188
Trimmed EI (10–90%)	0.010	0.125	-0.236	0.256
Trimmed Hurst (10–90%)	0.018	0.045	-0.070	0.107
Trimmed EI & Hurst (intersection)	0.081	0.112	-0.139	0.300

Table S4. EI fixed-effect estimates (Estimate ± 95% CI) for the full sample and each trimmed sample.

Metabolites

sLASER

All metabolite concentrations and Cramer-Rao lower bounds from semi-LASER are reported in Table S5.

	Rest Conc	Rest CRLB	Movie Conc	Movie CRLB
Asc	0.34 ± 0.29	239.72 ± 364.52	0.34 ± 0.24	194.39 ± 307.8
Asp	3.49 ± 0.44	9.56 ± 1.34	3.39 ± 0.43	9.72 ± 1.81
Cr	2.04 ± 0.62	16.33 ± 7.65	2.1 ± 0.49	15.22 ± 5.25
GABA	0.69 ± 0.41	52.78 ± 30.69	0.67 ± 0.32	49.06 ± 28.51
GPC	0.92 ± 0.11	12.11 ± 4.83	0.9 ± 0.11	13.56 ± 4.23
GSH	1.3 ± 0.18	7.61 ± 1.38	1.32 ± 0.13	7.44 ± 1.04
Gln	2.82 ± 0.58	10.11 ± 1.78	2.93 ± 0.47	9.72 ± 1.02
Glu	7.02 ± 0.71	4.33 ± 0.49	7.11 ± 0.62	4.17 ± 0.51
mI	4.77 ± 0.34	3 ± 0	4.8 ± 0.29	3 ± 0
Lac	0.32 ± 0.13	62.78 ± 38.94	0.28 ± 0.16	182.33 ± 311.77
NAA	11.88 ± 1.11	1.94 ± 0.24	11.83 ± 1.03	1.83 ± 0.38
NAAG	0 ± 0	999 ± 0	0.07 ± 0.22	890.78 ± 314.99
PCh	0.16 ± 0.12	292.5 ± 394.55	0.18 ± 0.1	179.89 ± 299.51
PCr	5.68 ± 0.84	5.83 ± 1.04	5.7 ± 0.69	6.11 ± 1.23
PE	1.67 ± 0.49	31.28 ± 11.85	1.71 ± 0.53	29.5 ± 8.58
sI	0.13 ± 0.12	198.61 ± 369.4	0.13 ± 0.11	127.56 ± 244.45
Tau	1.63 ± 0.35	15.44 ± 3.2	1.65 ± 0.3	15.06 ± 3.23
CrCH2	0.26 ± 0.28	433.94 ± 469.27	0.24 ± 0.2	285.11 ± 397.57

	Rest Conc	Rest CRLB	Movie Conc	Movie CRLB
tCho	1.07 ± 0.09	3.72 ± 0.46	1.07 ± 0.09	3.56 ± 0.62
tCr	7.72 ± 0.41	1.44 ± 0.51	7.8 ± 0.37	1.39 ± 0.5
tNAA	11.88 ± 1.11	1.94 ± 0.24	11.91 ± 1.04	1.83 ± 0.38
Glx	9.84 ± 1.06	4.39 ± 0.5	10.04 ± 0.79	4.17 ± 0.51
Lip13a	18.63 ± 7.85	76.06 ± 230.43	20 ± 7.47	21.78 ± 6.48
Lip13b	0.07 ± 0.3	944.39 ± 231.7	0 ± 0	999 ± 0
Lip09	4.91 ± 2.05	25 ± 9.44	5.22 ± 2.04	27 ± 10.96
MM09	3.1 ± 2.15	112.67 ± 226.41	3.03 ± 1.5	103.56 ± 227.35
Lip20	3.64 ± 1.34	15.5 ± 4.26	4.05 ± 1	14.44 ± 3.07
MM20	3.88 ± 3.43	242.11 ± 383.63	3.41 ± 2.16	143 ± 262.51
MM12	0.95 ± 0.93	316.28 ± 389.01	0.99 ± 0.97	333.78 ± 425.72
MM14	4.45 ± 2.14	34.39 ± 21.5	5.11 ± 2.63	35.67 ± 25.59
MM17	2.56 ± 1.84	246.39 ± 414.02	2.69 ± 1.1	39.89 ± 31.22

Table S5. ***All metabolite concentrations and Cramer-Rao lower bounds from semi-LASER.*** *Concentration values are in millimoles; Cramer-Rao lower bounds are in percentages. Conc = concentration; CRLB = Cramer-Rao lower bounds.*

MEGA-PRESS

All metabolite concentrations and Cramer-Rao lower bounds from J-edited MEGA-PRESS are reported in Table S6.

	Rest Conc	Rest CRLB	Movie Conc	Movie CRLB
GABA	1.64 ± 0.36	16.61 ± 5.51	1.55 ± 0.43	18.5 ± 5.86
GSH	2.41 ± 0.6	4.72 ± 0.57	2.17 ± 0.62	5.06 ± 0.94
Gln	2.59 ± 0.95	74.56 ± 230.78	2.4 ± 0.56	24.11 ± 9.54
Glu	12.41 ± 1.76	4.06 ± 0.64	12.11 ± 1.65	4.33 ± 0.77
NAA	16.47 ± 1.49	9.94 ± 2.21	16.29 ± 2.01	10.39 ± 1.5
NAAG	1.8 ± 0.57	16.61 ± 5.51	2.02 ± 1.07	18.5 ± 5.86
tNAA	18.27 ± 1.41	4.72 ± 0.57	18.31 ± 1.65	5.06 ± 0.94
Glx	15 ± 1.51	74.56 ± 230.78	14.51 ± 1.57	24.11 ± 9.54
MM09	3.84 ± 0.77	4.06 ± 0.64	3.69 ± 0.49	4.33 ± 0.77
GABAplus	5.48 ± 0.77	9.94 ± 2.21	5.23 ± 0.71	10.39 ± 1.5

Table S6. ***All metabolite concentrations and Cramer-Rao lower bounds from J-edited MEGA-PRESS.*** *Concentration values are in millimoles; Cramer-Rao lower bounds are in percentages. Conc = concentration; CRLB = Cramer-Rao lower bounds.*

Data quality

Data quality correlations for Hurst with mean framewise displacement, Glx and GABA+ with FWHM, and Glx and GABA+ with frequency shift are shown in Figure S2.

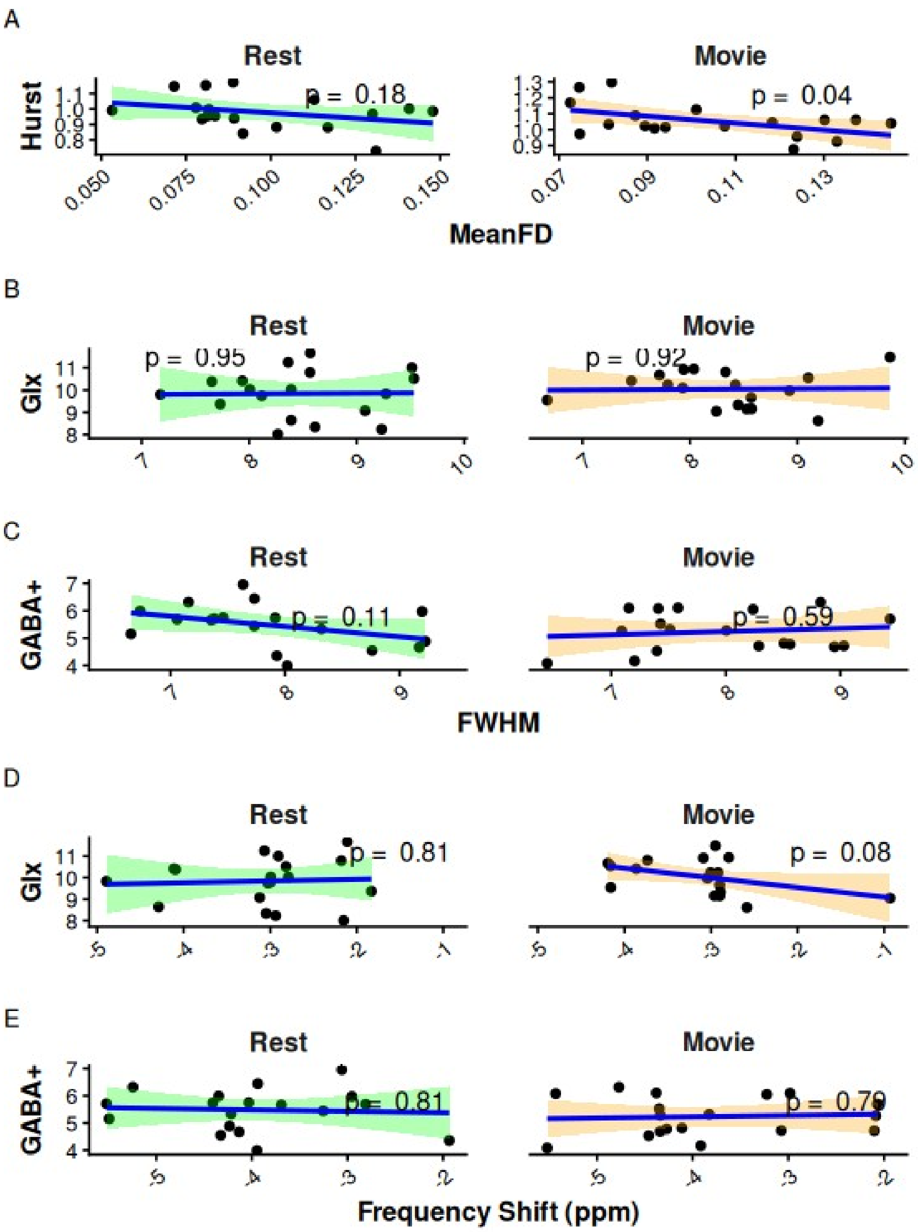

Figure S2. ***Data quality correlations.*** *A) Correlation plots of H vs. mean FD (mm) for rest (green) and movie (orange). B) and C) Correlation plots of Glx (B) and GABA+ (C) vs. water FWHM (Hz) for rest (green) and movie (orange). D) and E) Correlation plots of Glx (B) and GABA+ (C) vs. frequency shift (ppm) for rest (green) and movie (orange).*

Rest vs movie FWHM comparisons (paired t-tests) are provided in Table S7.

Seq	m. rest	m. movie	m. diff	sd diff	t	p	CIlow	CIhigh
MEGA	7.86	7.95	0.09	0.51	0.75	0.46	-0.16	0.34
sLASER	8.47	8.32	-0.15	0.49	-1.28	0.22	-0.39	0.10

*Table S7***.** *Rest vs. movie FWHM comparison (paired t-tests) for each spectroscopy sequence. Seq = sequence; m. = mean; sd = standard deviation; t = t-value; p = p-value; CIlow = lower confidence interval; CIhigh = higher confidence interval.*

Rest vs movie FWHM comparisons (paired t-tests) are provided in Table S8.

Seq	m. rest	m. movie	m. diff	sd diff	t	p	CIlow	CIhigh
MEGA	101.05	98.75	-2.30	9.39	-1.04	0.31	-6.97	2.36
sLASER	147.61	140.60	-7.01	22.84	-1.30	0.21	-18.37	4.34

*Table S8***.** *Rest vs. movie CR-SNR comparison (paired t-tests) for each spectroscopy sequence. Seq = sequence; m. = mean; sd = standard deviation; t = t-value; p = p-value; CIlow = lower confidence interval; CIhigh = higher confidence interval.*

Subject-level FWHM and CR-SNR values for each sequence (Rest, Movie, and Movie–Rest difference) are provided in Table S9.

Sub	Seq	R FWHM	M FWHM	Diff FWHM	R SNR	M SNR	Diff SNR
1	sLASER	8.36	8.04	-0.33	135.58	125.26	-10.32
1	MEGA	7.38	7.41	0.03	91.29	92.46	1.17
2	sLASER	8.57	7.72	-0.85	128.76	147.92	19.16
2	MEGA	7.73	7.15	-0.58	82.16	84.05	1.89
4	sLASER	8.61	8.93	0.31	162.00	132.33	-29.67
4	MEGA	9.23	8.56	-0.66	98.51	103.83	5.31
6	sLASER	8.01	7.94	-0.06	130.77	152.24	21.47
6	MEGA	7.35	7.58	0.23	128.02	122.19	-5.83
7	sLASER	9.08	9.10	0.02	112.42	99.45	-12.97
7	MEGA	9.17	8.95	-0.22	75.46	84.31	8.85
9	sLASER	8.27	8.53	0.26	172.88	161.56	-11.32
9	MEGA	8.02	8.51	0.49	115.78	108.07	-7.71
10	sLASER	7.65	7.46	-0.20	168.43	173.85	5.41
10	MEGA	6.66	7.20	0.55	115.75	103.79	-11.96
11	sLASER	9.53	8.57	-0.96	157.59	136.11	-21.48
11	MEGA	7.73	8.83	1.10	101.11	87.58	-13.52
12	sLASER	8.39	7.79	-0.60	180.44	136.71	-43.73
12	MEGA	7.46	7.43	-0.03	113.05	112.73	-0.32
13	sLASER	7.73	8.45	0.72	158.00	207.77	49.78
13	MEGA	7.06	7.09	0.03	147.40	147.57	0.17
15	sLASER	9.27	8.25	-1.03	161.54	135.97	-25.57
15	MEGA	7.93	7.40	-0.53	105.05	124.51	19.47
16	sLASER	9.23	9.19	-0.04	155.48	135.41	-20.07
16	MEGA	8.32	8.29	-0.03	102.73	97.17	-5.55
18	sLASER	8.57	8.33	-0.24	116.65	117.28	0.62
18	MEGA	7.91	7.51	-0.40	75.65	83.37	7.71
19	sLASER	8.39	8.57	0.18	169.98	145.07	-24.92
19	MEGA	7.63	8.01	0.37	126.17	107.86	-18.31
20	sLASER	7.94	7.93	0.00	146.29	156.69	10.40
20	MEGA	7.16	8.24	1.08	100.81	85.99	-14.82
22	sLASER	9.51	9.86	0.35	121.74	96.63	-25.11
22	MEGA	9.20	9.43	0.24	60.44	60.70	0.26
24	sLASER	7.18	6.67	-0.50	138.38	121.95	-16.43
24	MEGA	6.73	6.44	-0.29	85.78	83.25	-2.53

26	sLASER	8.12	8.42	0.31	140.13	148.61	8.48
26	MEGA	8.76	9.03	0.27	93.80	88.04	-5.76

Table S9. Subject-level FWHM and CR-SNR values for each sequence (Rest, Movie, and Movie–Rest difference). Sub = Subject; Seq = Sequence; R = Rest; M = Movie; Diff = Difference.

Plots of individual water FWHM changes between rest and movie-watching conditions for both MEGA-PRESS and sLASER are shown in Figure S3.

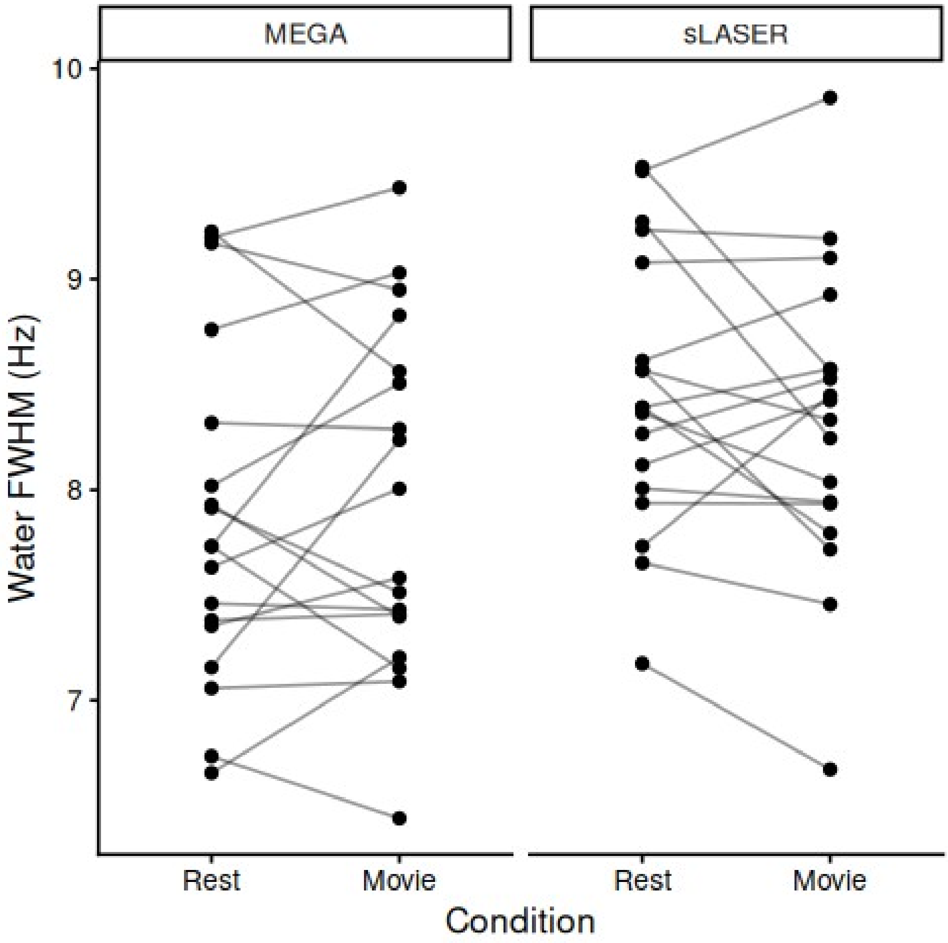

Figure S3. Plots of individual water full-width half-maximum changes between rest and movie-watching conditions for both MEGA-PRESS and sLASER

Frequency shift between rest and movie are shown in Figure S4.

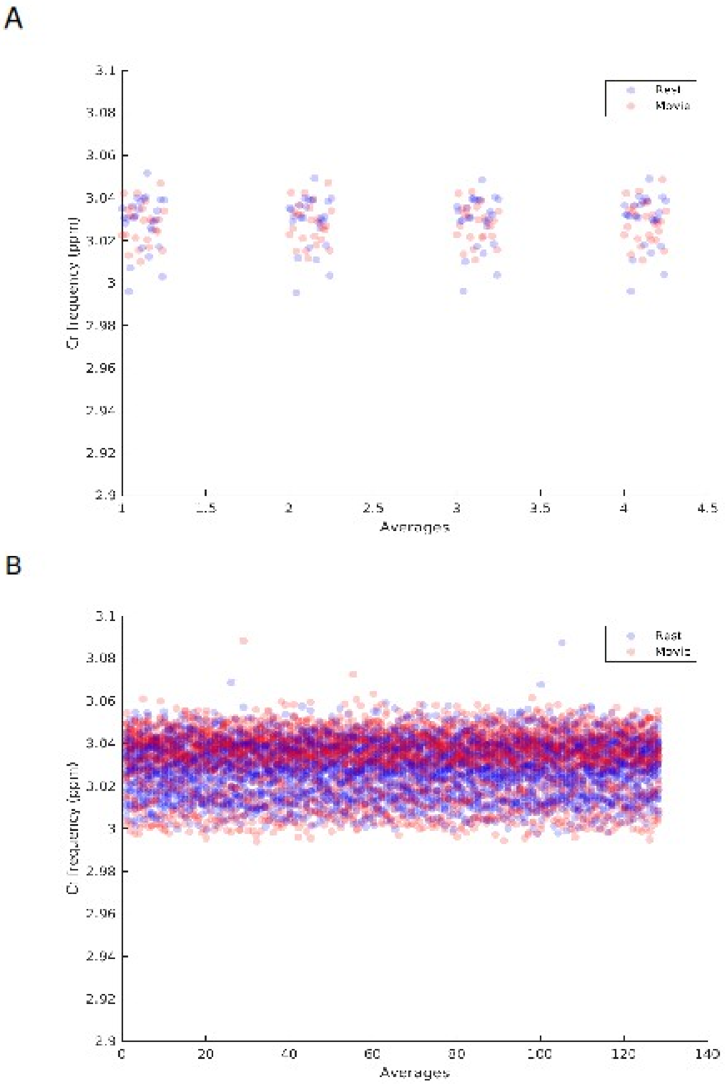

Figure S4. ***Frequency shift between rest and movie.*** *Overlay of all participants' frequency drift (ppm) plots during rest (blue) and movie (red) for A) semi-LASER and B) MEGA-PRESS. Note, the GE semi-LASER sequence averaged all transients (128) into groups of 4, each with 32 transients, before being saved as raw P-file.*

Gannet Results

An example fit result from Gannet of a subject at rest is shown in Figure S5.

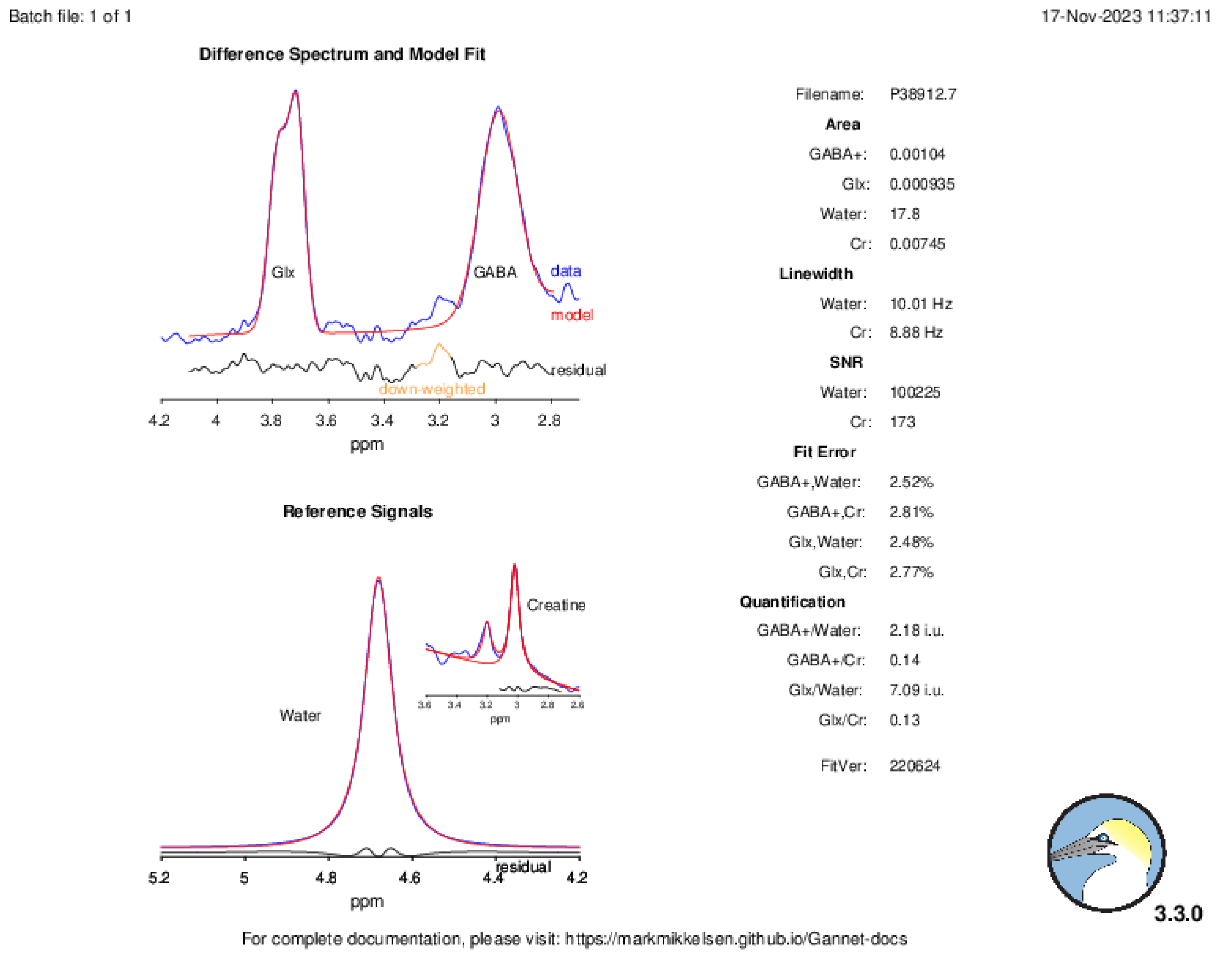

*Figure S5. **Gannet Fit.** Sample Gannet fit result from a subject a rest. Glx (3.75ppm) and GABA (3) data (blue) and model fit (red) are shown top left. Water and creatine reference signals are shown bottom left. Table on right shows GABA+ and Glx values before tissue-correction.*

To examine the relationship between H and E:I, we ran a linear mixed-effects model. The model's total explanatory power was substantial (conditional $r^2 = 0.64$), with fixed effects (marginal r^2) equal to 0.23. The model's intercept, corresponding to EI = 0, Condition = Movie, $FWHM_{MEGA\text{-}PRESS} = 0$, $FreqShift_{MEGA\text{-}PRESS} = 0$, and meanFD = 0, was 1.2 (95% CI [0.66, 1.7], $t(26) = 4.64$, $p < 0.01$). Within this model, only the effect of Condition [Rest] was statistically significant (beta = -0.08, 95% CI [-0.13, -0.03], $t(26) = -3.32$, $p < 0.01$). The effect of E:I was statistically non-significant (beta = -0.07, 95% CI [-0.21, 0.06], $t(26) = -1.04$, $p = 0.31$); along with $FWHM_{MEGA\text{-}PRESS}$ (beta = 0.03, 95% CI [-0.03, 0.08], $t(26) = 0.96$, $p = 0.35$); $FreqShift_{MEGA\text{-}PRESS}$ (beta = 0.01, 95% CI [-0.02, 0.05], $t(26) = 0.63$, $p = 0.53$); and meanFD (beta = -0.76, 95% CI [-2.34, 0.82], $t(26) = -0.95$, $p = 0.35$).

Mean ± sd of metabolites and E:I when analyzed using Gannet, during rest and movie, are reported in Table S10. Boxplots between rest and movie can be seen in Figure S6. Neither Glx nor GABA+ were different between movie and rest conditions. E:I ratio did not change between conditions either.

	Rest	Movie	p-value
**Glx**	10.84 ± 1.06	10.72 ± 0.68	0.43
**GABA+**	3.59 ± 0.38	3.53 ± 0.36	0.33
**E:I Ratio**	1.82 ± 0.25	1.94 ± 0.25	0.08

Table S10. ***Summary of main metabolite results using Gannet***

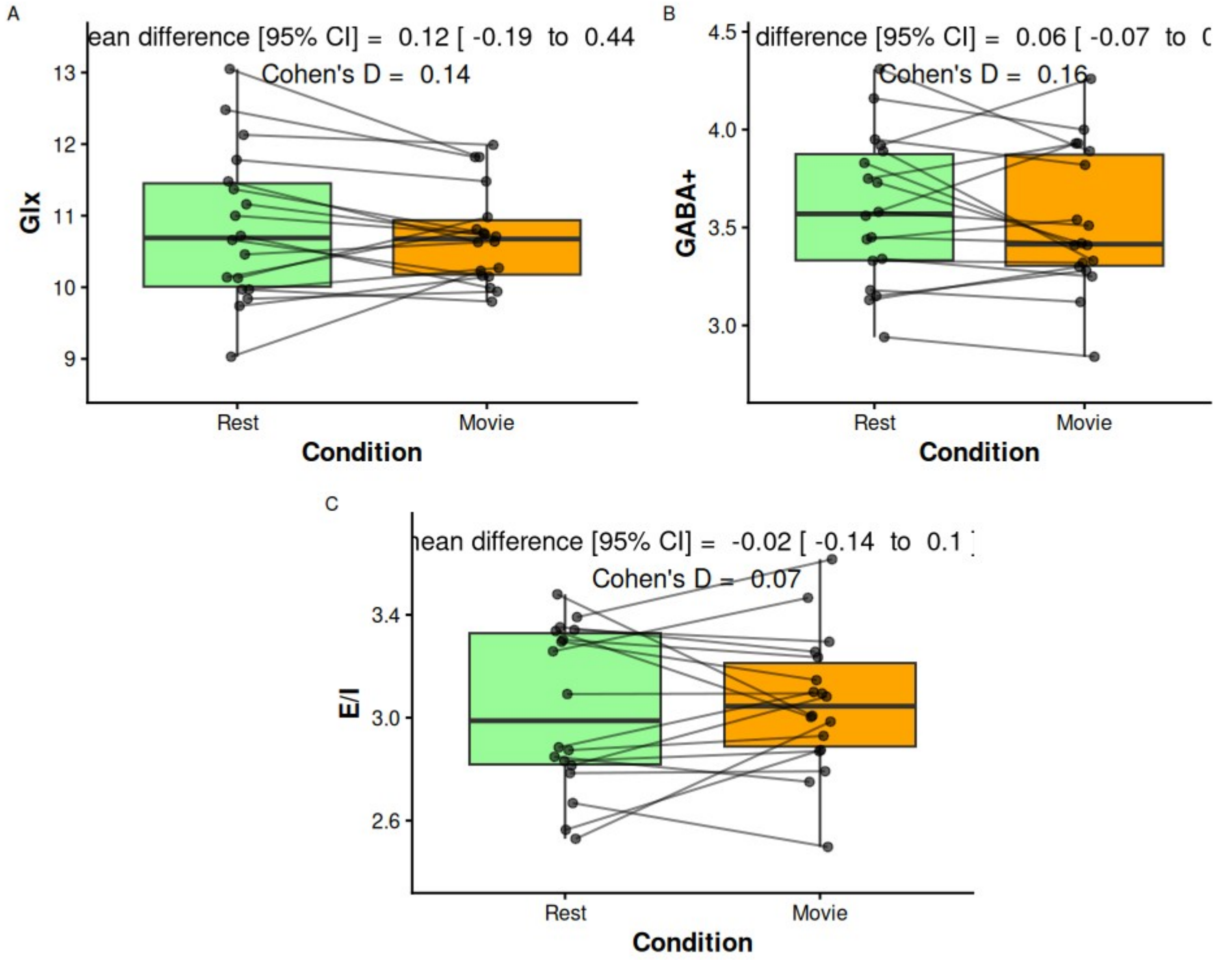

*Figure S6***. Paired comparison of metabolite values analyzed using Gannet during rest (green) and movie (orange) conditions.** *A) Glx (mM); B) GABA+ (mM); and C) E:I. Paired dots represent the same participant across conditions. Mean difference with 95% confidence intervals, and as Cohen's D are reported at the top of each plot."*

H was not found to correlate with Glx (Rest: r = 0, p = 0.99; Movie: r = -0.27, p = 0.28), GABA+ (Rest: r = 0.02, p = 0.93; Movie: r = 0.11, p = 0.67), or E:I (Rest: r = -0.04, p = 0.89; Movie: r = -0.32, p = 0.2) (Figure S7).

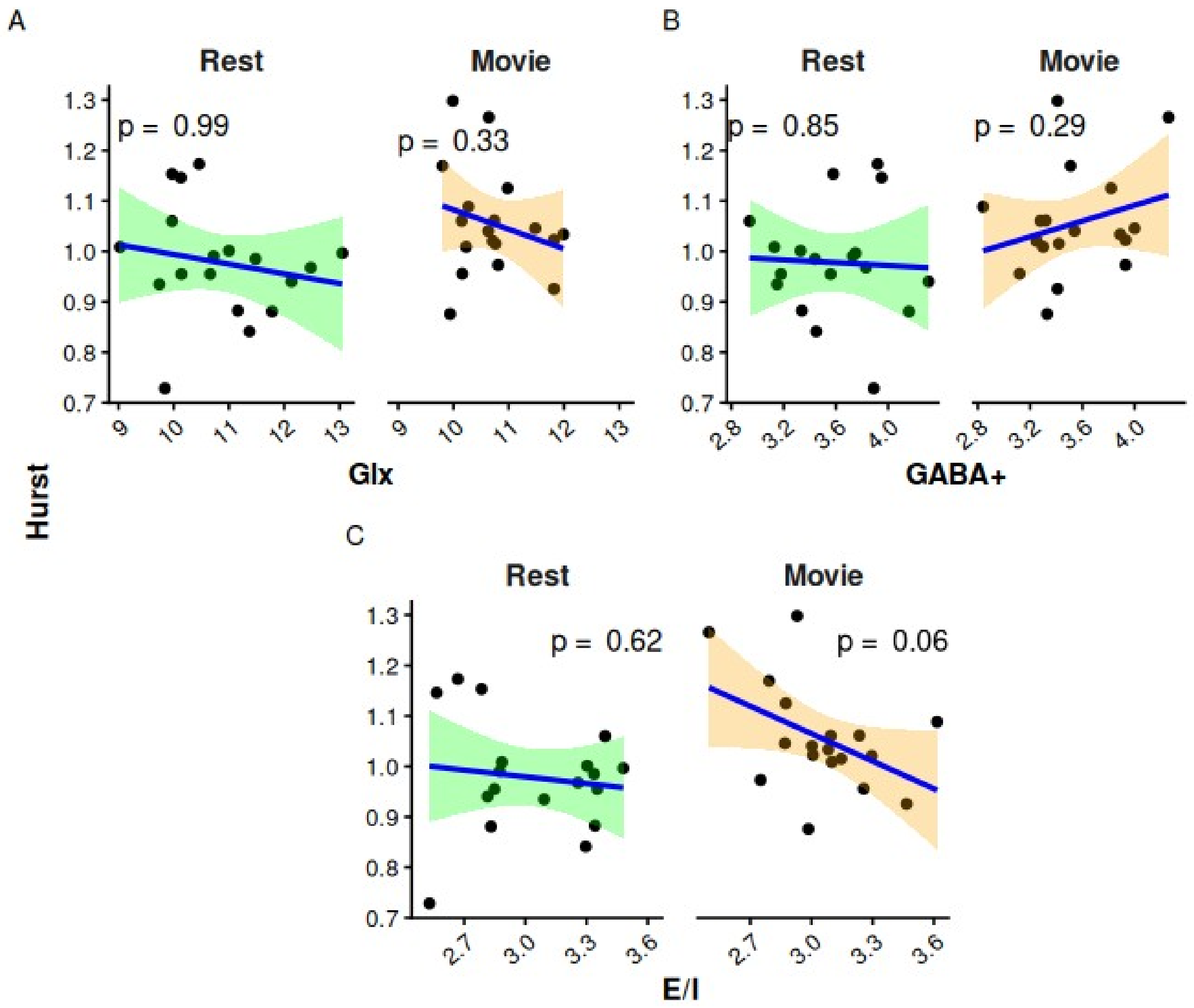

Figure S7. ***Scatter plots of H vs. metabolites when analyzed using Gannet.*** *A) Glx (mM); B) GABA+ (mM); and C) E:I. Rest is in green, while movie is in orange. P-values are reported at the top of each plot.*

Glx estimates were not strongly correlated between sLASER and MEGA-PRESS (Rest $r = 0.06$, Movie $r = 0.25$), while mean absolute differences remained under 1 institutional units. Table S11 reports the paired summary statistics, and Figure S8 visualises the subject-level agreement.

This finding is consistent with several studies that have directly compared Glx measured from MEGA-PRESS "OFF" spectra, with 'gold standard' short-TE sequences (PRESS and sLASER), which have consistently reported poor agreement (Bell et al., 2021; Thomson et al., 2026; Van Veenendaal et al., 2018). Reasons for this include the fact that MEGA-PRESS uses a longer TE time (68 ms compared to sLASER's 35 ms) (Maddock et al., 2018); chemical shift displacement

error differences between the two sequences (Archibald et al., 2025); and scanner drift due to MEGA-PRESS being acquired on average 6.43 ± 1.67 (Rest) and 5.89 ± 0.95 minutes (Movie) after sLASER.

Cond	n	m. sLASER	m. MEGA	m. diff	sd diff	m.a. diff	r	p	CI_{low}	CI_{high}
Movie	18	10.04	10.72	-0.67	0.90	0.91	0.25	0.31	-0.24	0.64
Rest	18	9.84	10.84	-1.00	1.45	1.28	0.06	0.80	-0.42	0.51

Table S11. Agreement between sLASER- and MEGA-PRESS-derived Glx within each condition. Cond = condition; n = number of subjects; m. = mean; m.a. = mean absolute; r = Pearson correlation; p = p-value; CIlow = lower confidence interval; CIhigh = higher confidence interval.

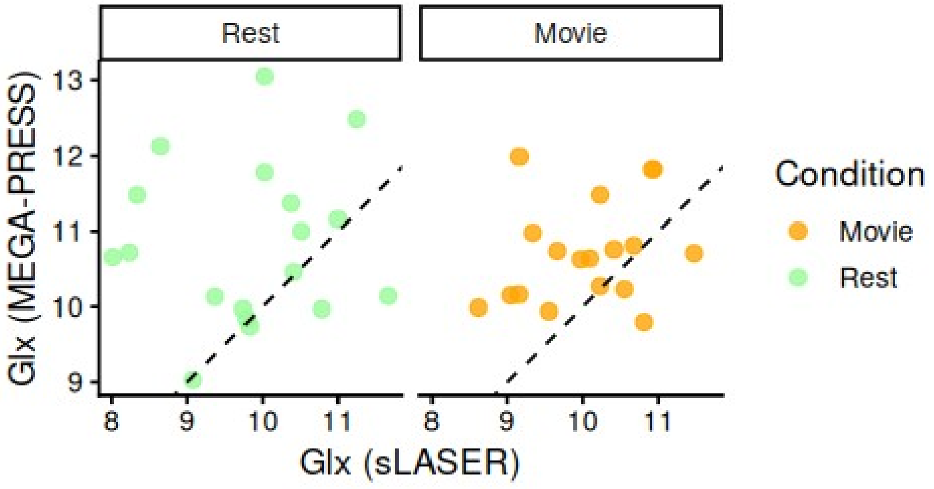

Figure S8. Glx from MEGA-PRESS plotted against Glx from sLASER, during both rest (green) and movie-watching (orange). Dashed line represents perfect agreement.

References

Archibald, J., Bouchard, A. E., Noeske, R., Shungu, D. C., & Mikkelsen, M. (2025). Test-retest reliability of multi-metabolite edited MRS at 3T using PRESS and sLASER. *BioRxiv: The Preprint Server for Biology*, 2025.06.07.657685. https://doi.org/10.1101/2025.06.07.657685

Bell, T., Boudes, E. S., Loo, R. S., Barker, G. J., Lythgoe, D. J., Edden, R. A. E., Lebel, R. M., Wilson, M., & Harris, A. D. (2021). In vivo Glx and Glu measurements from GABA-edited MRS at 3 T. *NMR in Biomedicine*, 34(5), e4245. https://doi.org/10.1002/nbm.4245

Maddock, R. J., Caton, M. D., & Ragland, J. D. (2018). Estimating glutamate and Glx from GABA-optimized MEGA-PRESS: Off-resonance but not difference spectra values correspond to PRESS values. *Psychiatry Research. Neuroimaging*, 279, 22–30. https://doi.org/10.1016/j.pscychresns.2018.07.003

Thomson, A. R., Hollestein, V., Goodwin, A., Fritz, A., Oakley, B., Murphy, D., Bullock, E., Demurie, E., Loth, E., Bussu, G., Roeyers, H., Yorke, I., Buitelaar, J. K., Koziel, J., Colomar, L., Krol, M. A., Bowdler, M., Herregods, N., Aggensteiner, P., … Puts, N. A. (2026). In Vivo Glx Measurements From GABA-Edited HERMES at 3 T Are Not Consistent With Those From Short-TE PRESS Across Scanners, Brain Regions, Diagnostic and Age Groups. *NMR in Biomedicine*, 39(1), e70171. https://doi.org/10.1002/nbm.70171
